# Unveiling Metabolic Signatures as Potential Biomarkers in Common Cancers: Insights from Lung, Breast, Colorectal, Liver, and Gastric Tumours

**DOI:** 10.3390/biom15101376

**Published:** 2025-09-28

**Authors:** Kha Wai Hon, Rakesh Naidu

**Affiliations:** Jeffrey Cheah School of Medicine and Health Sciences, Monash University Malaysia, Jalan Lagoon Selatan, Bandar Sunway 47500, Selangor, Malaysia; dominichon93@gmail.com

**Keywords:** biomarker, breast cancer, colorectal cancer, gastric cancer, lung cancer, liver cancer, metabolic proteins

## Abstract

Reprogramming is a hallmark of cancer, enabling tumour cells to sustain rapid proliferation, resist cell death, and adapt to hostile microenvironments. This review explores the expression profiles of key metabolic enzymes and transporters involved in glucose, amino acid, and lipid metabolism across the five most deadly cancers worldwide: lung, breast, colorectal, liver, and gastric cancers. Through a comparative analysis, we identify consistent upregulation of glycolytic enzymes such as LDHA, PKM2, and HK2, as well as nutrient transporters like GLUT1, ASCT2, and LAT1, which contribute to cancer progression, metastasis, and therapy resistance. The role of enzymes involved in glutaminolysis (e.g., GLS1, GDH), one-carbon metabolism (e.g., SHMT2, PHGDH), and fatty acid synthesis (e.g., FASN, ACLY) is also examined, with emphasis on their emerging relevance as diagnostic, prognostic, and predictive biomarkers. While several metabolic proteins show strong potential for clinical translation, only a few, such as tumour M2-pyruvate kinase (TuM2-PK) and serum LDH measurement, have progressed into clinical use or trials. This review addresses some of the challenges in biomarker development. Ultimately, our findings underscore the importance of metabolic proteins not only as functional drivers of malignancy but also as promising candidates for biomarker discovery. Advancing their clinical implementation could significantly enhance early detection, treatment stratification, and personalized oncology.

## 1. Introduction

Cancer is a leading global health challenge, accounting for a significant proportion of morbidity and mortality worldwide. According to the latest Global Cancer Statistics 2022, an estimated 20 million new cancer cases were diagnosed globally, highlighting its pervasive impact, with lung, breast, colorectal (CRC), prostate, stomach, liver and thyroid cancers ranking as the most common malignancies [1]. Cancer also remains a significant cause of mortality, contributing to approximately 9.7 million deaths globally in 2022 [1]. Lung cancer is the top contributor to cancer-related deaths (1.82 million deaths, 18.7% of total), followed by CRC (90,000 deaths, 9.3%), liver cancer (760,000 deaths, 7.8%), breast cancer (670,000 deaths, 6.9%), and stomach cancer (660,000 deaths, 6.8%) [1]. Projections indicate that the global cancer burden will continue to rise, with annual new cases expected to reach 35.3 million by 2050, which is almost an 80% increase from 2020 [2]. Additionally, 18.5 million cancer deaths are projected by 2050, which is an 89.7% increase from the 2022 estimate of 9.7 million [2]. These statistics and projections underscore the urgent need for improved diagnostic and therapeutic strategies, mainly by identifying reliable cancer biomarkers.

Cancer biomarkers are molecules that indicate the presence or progression of cancer, and their identification and application have significantly advanced cancer diagnosis, prognosis, and treatment. These biomarkers include proteins, DNA, RNA, metabolites, and cellular changes, which can be detected in body fluids, tissues, or cells [3,4,5]. The advent of high-throughput technologies such as next-generation sequencing (NGS), proteomics, and metabolomics has accelerated the discovery of novel biomarkers [6,7,8,9]. For example, NGS enables comprehensive analysis of genomic alterations, facilitating the identification of cancer-specific mutations like BRCA1/2 in breast and ovarian cancers, TP53 in multiple cancer types, KRAS in CRC and lung cancers, BRAF in melanoma and thyroid cancer, PIK3CA in breast cancer, and APC in CRC [10,11]. More importantly, cancer biomarkers based on metabolic enzymes, drug transporters, and metabolic proteins are gaining prominence due to their critical roles in tumour growth, metabolism, drug resistance, and therapy response [12,13,14]. These biomarkers are primarily associated with cancer-specific alterations in cellular metabolism (such as the Warburg effect, glutamine and lipid metabolism) and drug metabolism [12,13,14].

Metabolic reprogramming is a key hallmark of cancers, in which cancer cells adapt their metabolism to sustain rapid proliferation, resist stress, evade immune responses, and adapt to the tumour microenvironment [15]. One of the most well-known metabolic alterations in cancer cells is the Warburg effect, which refers to the preference of cancer cells for aerobic glycolysis, even in the presence of sufficient oxygen [16]. This shift from oxidative phosphorylation to glycolysis can provide cancer cells with rapid ATP production and rapid synthesis of intermediates, such as nucleotides, amino acids, and lipids, which are crucial for proliferation [16]. Additionally, mitochondrial metabolism still plays an essential role in the metabolic reprogramming of cancer cells by contributing to biosynthesis, redox balance, and suppression of apoptosis [17,18]. Cancer cells also demonstrate a pronounced preference for glutamine metabolism, which is an amino acid critical for replenishing tricarboxylic acid (TCA) cycle intermediates, providing nitrogen for biosynthetic pathways, and producing glutathione to manage reactive oxygen species (ROS) [19]. Cancer cells also tend to reprogram lipid metabolism by enhancing fatty acid synthesis and uptake to support membrane biosynthesis, energy storage and regulation of signalling pathways [20].

Redox homeostasis is often altered in the metabolic reprogramming of cancer cells, in which elevated metabolic activity and ROS production necessitate robust antioxidant systems, such as increased glutathione synthesis and upregulation of the pentose phosphate pathway (PPP), to maintain cellular survival under oxidative stress [21,22,23,24]. Similarly, the tumour microenvironment, which is usually hypoxic, further drives metabolic shifts through the activation of hypoxia-inducible factors (HIFs), which enhance glycolysis and lactate production while suppressing mitochondrial oxidative phosphorylation [25,26]. Key enzymes such as hexokinase 2 (HK2), pyruvate kinase M2 (PKM2), and glutaminase (GLS) regulate glycolysis, glutaminolysis, and biosynthetic pathways, while transporters like GLUT1 and ASCT2 mediate increased glucose and amino acid uptake, respectively, highlighting their roles in sustaining cancer cell metabolism [27,28,29,30,31,32]. Additionally, enzymes like fatty acid synthase (FASN) and glucose-6-phosphate dehydrogenase (G6PD) support lipid biosynthesis and antioxidant defence, respectively, to fulfil the biochemical demands of rapidly proliferating tumours [33,34,35].

Membrane transporters such as monocarboxylate transporters (MCTs) facilitate lactate export, contributing to an acidic microenvironment that promotes invasion and immune evasion, while drug transporters, including ABCC1, BCRP and P-gp, modulate the uptake and efflux of chemotherapeutic agents, impacting drug efficacy and resistance [12,36,37]. These molecules are frequently dysregulated in cancers, making them ideal biomarkers for early detection, prognosis, and therapeutic response monitoring. These metabolic components can be leveraged to identify cancer-specific signatures by targeting their specific expression patterns or functional activity, improving diagnostic precision and developing novel, metabolism-focused therapeutic strategies. Therefore, this review aims to provide insights into the discovery of metabolic enzymes, proteins, and transporters as potential biomarkers for the top five most deadly cancers, including lung, CRC, breast, liver and stomach.

## 2. The Warburg Effect and Glucose Metabolism

The Warburg effect is defined by the preference of cancer cells for aerobic glycolysis over oxidative phosphorylation. Several key glycolytic enzymes that regulate glucose metabolism and associated pathways have been widely reported in cancers, including ALDOA, ENO1, HK2, LDH, PFK1, PGAM1, PGK1, PKM2 and TPI1 [38,39,40,41]. Lactate dehydrogenase (LDH) is the most widely studied in cancer diagnosis, while different isoforms could be associated with distinct responses to cancer treatment [42]. Lactate dehydrogenase A (LDHA) converts pyruvate to lactate, regenerating NAD+ and supporting the production of lactate, which acidifies the tumour microenvironment to promote invasion and immune evasion [43]. Lactate dehydrogenase B (LDHB) could be dysregulated in specific cancer types depending on the glycolytic requirements, while most of the time, LDHB is silenced by promoter methylation [44,45,46].

Aldolase A (ALDOA) is a key glycolytic enzyme responsible for the cleavage of fructose-1,6-bisphosphate into glyceraldehyde-3-phosphate and dihydroxyacetone phosphate [47]. Beyond its metabolic role, ALDOA has been increasingly recognised for its involvement in cancer progression by promoting EMT, metastasis and tumour progression [48,49,50]. ALDOA is often upregulated in cancers, particularly those aggressive phenotypes with high glycolytic activity and associated with poor prognosis [48,50,51,52,53]. Alpha enolase (ENO1) catalyses the interconversion of 2-phosphoglycerate to phosphoenolpyruvate in both gluconeogenesis and glycolytic metabolic pathways [54]. In cancers, ENO1 has a non-metabolic function as a plasminogen receptor in the tumour cell surface, enhancing proliferation, migration, invasion, and metastasis [55,56]. Overexpression of ENO1 is mainly associated with chemoresistance, poor prognosis, reduced survival and stemness in cancers [55,57,58,59,60,61].

Hexokinase 2 (HK2) catalyses the initial phosphorylation of glucose to sustain glycolytic flux into cancer cells, while its overexpression enhances glycolytic flux, supporting drug resistance and tumour growth besides associated with poor prognosis [14,54,62,63,64,65,66]. Phosphofructokinase-1 (PFK1) controls the rate-limiting step, converting fructose-6-phosphate to fructose-1,6-bisphosphate [38]. Elevated PFK1 expression, particularly the PFKP isoform, is often linked to enhanced glycolysis, tumour growth, and resistance to therapies, making it a potential target for cancer treatment strategies [67,68]. Moreover, phosphoglycerate kinase 1 (PGK1) catalyses the reversible transfer of a phosphate group from 1,3-bisphosphoglycerate (1,3-BPG) to ADP, producing 3-phosphoglycerate (3-PG) and ATP in the first rate-limiting step of glycolysis [54]. It has non-glycolytic roles in tumour progression by promoting angiogenesis and metastasis, while its overexpression is associated with poor prognosis and drug resistance [69,70,71]. Phosphoglycerate mutase 1 (PGAM1) is a glycolytic enzyme that catalyses the conversion of 3-phosphoglycerate (3-PG) to 2-phosphoglycerate (2-PG).

Pyruvate kinase M2 (PKM2) is a cancer-specific isoform that facilitates the conversion of phosphoenolpyruvate (PEP) to pyruvate, with its less efficient activity allowing glycolytic intermediates to accumulate for biosynthesis [72]. Overexpression of PKM2 isoform in cancers is mainly associated with treatment response, metastasis, poor prognosis and reduced survival, making it a promising biomarker for diagnosis and treatment monitoring [73,74,75]. Triose phosphate isomerase (TPI) catalyses the interconversion between dihydroxyacetone phosphate and glyceraldehyde-3-phosphate in glycolysis and gluconeogenesis [76]. Elevated TPI levels in patient samples correlate with poor prognosis, tumour proliferation, migration, and invasion [77,78,79,80]. In addition, glucose transporters (GLUTs) play a crucial role in the Warburg effect by increasing glucose uptake for glycolysis and anabolic processes [54]. The most relevant GLUTs in cancer include GLUT1 (SLC2A1), GLUT3 (SLC2A3) and GLUT4 (SLC2A4), which are highly expressed in breast, CRC and lung cancers, with a high affinity for glucose and commonly associated with poor prognosis and therapy resistance [32,81,82]. The upregulation of glycolytic enzymes and glucose transporters collectively enables the metabolic reprogramming necessary for rapid energy production and biosynthesis to support cancer cell proliferation, stress adaptation, and tumour progression. However, the expression levels of key glycolytic enzymes vary across different cancer types, reflecting the metabolic adaptations of various tumour types to support their growth and survival.

### 2.1. Breast Cancer and Glucose Metabolism

In breast cancer, elevated LDHA levels have been detected in cancer tissue and patient serum, suggesting that LDHA could be released from breast cancer cells into the body circulation and remain stable for clinical detection [83,84,85]. In metastatic breast cancer, LDHA overexpression in patient serum was correlated with poor prognosis and unfavourable survival after first-line chemotherapy treatment, which could be an independent prognostic biomarker [83]. Patients with basal-like/triple negative BC (TNBC) expressed increased levels of LDHA and LDHB in serum measurement and tissue microarray analysis, which were significantly associated with shorter survival, poor prognosis, pathological complete response (pCR) to neoadjuvant chemotherapy and cancer relapse, suggesting the potential of LDHA and LDHB as biomarkers for treatment monitoring [86,87]. Dong et al. also reported that LDHA overexpression in cancer tissue via immunohistochemistry and serum LDH status was positively correlated with a higher incidence of brain metastasis among TNBC patients, suggesting the potential of LDHA as a prognostic factor in TNBC [88]. Collectively, elevated levels of LDHA and LDHB in breast cancer tissue could be developed into tissue-based assays. The measurement of LDHA in patient serum may facilitate less invasive testing methods for routine monitoring.

Both PKM2 and HK2 were significantly upregulated in breast cancer tissues compared to adjacent normal tissues [64,89,90]. Elevated expression levels of these glycolytic enzymes have been associated with advanced clinical stages, positive lymph node involvement, and distant metastasis [64,89,90]. Based on microarray data and immunohistochemical (IHC) analysis, HK2 protein expression was significantly correlated with HIF-1α immunoreactivity, Ki67 status and cancer recurrence in breast cancer patients [64]. Similarly, PKM2 overexpression was observed in tissue samples of various breast cancer subtypes, including TNBC, luminal, and HER2-positive cancers, significantly associated with poor prognosis, EMT and increased chemosensitivity to epirubicin and 5-fluorouracil [89,90]. These findings suggest that PKM2 and HK2 play crucial roles in breast cancer progression and may serve as potential biomarkers or therapeutic targets. Additionally, PFK1 levels were higher in breast cancer tissues than in adjacent non-cancerous tissues, with a prominent shift in isoform expression [91]. Breast cancer tissues predominantly express the platelet-type isoform (PFKP), whereas adjacent tissues mainly express the liver-type isoform (PFKL) [91]. This switch was associated with increased glycolytic activity in tumour cells, highlighting the potential role of cancer-specific PFK1 isoform, predominantly PFKP, in clinical diagnosis. Coelho et al. also demonstrated that the intracellular distribution and enzymatic activities of HK2 and PFK1 were positively correlated with the aggressiveness and invasiveness of different breast carcinoma subtypes, highlighting the great potential of these enzymes as prognostic biomarkers in breast cancer [92].

IHC analysis revealed that PGAM1 was significantly overexpressed in tissue samples of invasive ductal breast carcinomas and associated with poor overall survival [93]. PGAM1 exhibited metabolic and non-metabolic roles in breast cancer cells, in which it promoted cell proliferation by upregulating glycolysis besides interacting with cytoskeletal protein ACTA2 to induce cell migration and metastasis [93]. Furthermore, the expression of glucose transporters, especially GLUT1, has been extensively investigated in breast cancer samples due to their role in facilitating increased glucose uptake in metabolic reprogramming. Previous studies have shown that GLUT1 was overexpressed in invasive ductal carcinoma and TNBC based on IHC staining [94,95,96]. This overexpression was significantly associated with higher nuclear grade and negative status for EGFR, HER2, oestrogen and progesterone receptors [94,96]. Patients with GLUT1-positive tumours had shorter disease-free and overall survival periods, suggesting that GLUT1 expression correlates with more aggressive tumour behaviour and poorer prognosis [94].

Another IHC study by Sun et al. reported that GLUT1 was significantly overexpressed in the tissue of invasive ductal breast carcinoma (IDBC) with type 2 diabetes mellitus (T2DM) compared to those without T2DM, suggesting enhanced glucose uptake and metabolic reprogramming in IDC [97]. GLUT1 overexpression was closely associated with the upregulation of matrix metalloproteinases MMP2 and MMP9 in IDBC tissue, which is crucial for extracellular matrix degradation and tumour cell invasion [97]. This study establishes a mechanistic link between glucose metabolism and tumour invasiveness, where GLUT1 overexpression promotes a more aggressive cancer phenotype in breast cancer [97]. These findings highlight the importance of GLUT1 as a potential target for future therapeutic development to reduce tumour invasiveness and metastasis in breast cancer patients with metabolic disorders such as diabetes.

Pinheiro et al. investigated the expression profiles of GLUT1 and carbonic anhydrase IX (CAIX) in breast cancer tissue via IHC and their correlation with adverse prognostic factors, particularly monocarboxylate transporter 1 (MCT1) overexpression [98]. GLUT1 and CAIX were significantly upregulated in aggressive breast cancer subtypes and strongly associated with poor prognostic factors, including high tumour grade, hypoxic tumour microenvironment, and increased metabolic reprogramming [98]. This study highlights a positive correlation between GLUT1, CAIX, and MCT1 overexpression, suggesting a cooperative role in tumour progression by facilitating glycolytic metabolism and acid-base balance in hypoxic conditions. These findings underscore the potential of GLUT1, CAIX, and MCT1 as prognostic biomarkers and possible therapeutic targets in breast cancer. Their co-expression may help refine patient stratification, guide treatment decisions, and support the development of novel metabolic inhibitors to improve outcomes in aggressive breast cancer cases.

HER2-positive (HER2^+^) breast cancer is another highly aggressive subtype and is commonly associated with poor prognosis [99]. Previously, Wang et al. investigated the role of sodium-dependent glucose transporter 1 (SGLT1) in HER2^+^ breast cancer, focusing on its stabilisation by HER2 and its involvement in oncogenic signalling [100]. SGLT1 and GLUT1 are involved in glucose transport but differ in their mechanisms, functions, and expression patterns. SGLT1 is an active transporter that uses sodium gradients to import glucose into cells, especially under low-glucose conditions actively [101]. In contrast, GLUT1 is a passive transporter that allows glucose to move down its concentration gradient, supporting rapid glucose uptake in highly glycolytic tumours [54]. GLUT1 is more commonly overexpressed in cancer cells, whereas SGLT1 expression in cancers is little known. SGLT1 was significantly overexpressed in HER2^+^ breast cancer tissues as confirmed via IHC staining, which positively correlated with HER2 status, lymph node metastasis, shorter disease-free survival and poor overall survival [100]. Further in-depth analysis showed that HER2 stabilisation prevented SGLT1 degradation, maintaining intracellular glucose levels critical for cancer cell survival [100]. Inhibition of HER2 using targeted inhibitors reduced SGLT1 expression in HER2^+^ breast cancer cells, while suppressing SGLT1 function using phlorizin or HER2 inhibitors led to a significant decrease in glycolytic activity with downregulation of PI3K/Akt/mTOR signalling, reducing breast cancer cell proliferation [100]. This study identified SGLT1 as a key metabolic and prognostic marker in HER2^+^ breast cancer, linking glucose transport with oncogenic signalling.

The expression of other glucose transporters has been explored in breast cancer studies. Krzeslak et al. examined GLUT1 and GLUT3 protein expression in breast cancer tissue using Western blot, which reported the presence of GLUT1 in 48.7% and GLUT3 in 22.8% of tumour samples [102]. Though not statistically significant, GLUT1 expression was more prevalent in poorly differentiated tumours (grades 2 and 3) than in well-differentiated ones [102]. Compared to normal breast tissue, GLUT1 was detected in 22.2% and GLUT3 in 16.7% of samples, suggesting increased expression in cancerous tissues. These findings highlight GLUT1 as a more dominant glucose transporter in breast cancer, while GLUT3 may play a more selective role, potentially linked to aggressive tumour phenotypes, warranting further investigation.

Monocarboxylate transporters (MCTs) are integral membrane proteins that facilitate the transmembrane transport of monocarboxylates, such as lactate and pyruvate. MCT1, MCT2, and MCT4 expression patterns have been investigated in breast cancer patient samples to understand their roles in tumour progression and potential as therapeutic targets. Based on an IHC study using high-density tissue microarray, MCT1 (SLC16A1) was overexpressed in glycolytic breast tumours, which was associated with increased glycolytic activity and poor prognosis in breast cancer patients [103]. Inhibition of MCT1 impairs the proliferation of glycolytic breast cancer cells by disrupting pyruvate export, leading to enhanced oxidative metabolism and reduced tumour growth [103]. Similarly, Johnson et al. investigated the MCT1 expression in different subtypes of IDC using tissue microarrays [104]. High MCT1 expression was observed in TNBC cases, compared to ER+ and/or PR+ and HER2^+^ tumours [104]. MCT1 overexpression was positively correlated with large tumour size, high-grade tumours, shorter progression-free survival, and cancer recurrence, irrespective of breast cancer subtypes [104]. MCT1 could be a prognostic biomarker in IDC.

IHC analyses have demonstrated MCT2 expression in breast carcinoma samples, with some evidence suggesting a correlation between high MCT2 expression and poor patient prognosis. Elevated MCT2 (SLC16A7) levels were associated with increased expression of interleukin-1β (IL-1β) and lipocalin-2 (LCN2), both of which are linked to tumour progression and adverse outcomes [105]. Combined high expression of MCT2 with IL-1β or LCN2 has been proposed as a potential biomarker for poor prognosis in breast cancer patients [105]. Meanwhile, MCT4 (SLC16A3) was upregulated in breast cancer tissue in IHC analysis, particularly TNBC subtypes, and associated with high tumour grade and poor prognosis [82]. Functional studies have shown that the PI3K regulates the MCT4–Akt signalling pathway to support breast cancer cell survival under hypoxic conditions, contributing to tumour growth and disease progression [82]. The authors also proposed that tumoral MCT4 expression could serve as an independent prognostic survival factor for TNBCs and node-negative breast cancer [82]. The differential expression patterns and functional roles of MCT1, MCT2 and MCT4 highlight their potential as prognostic biomarkers and therapeutic targets in breast cancer. In summary, evidence suggests that GLUT1, HK2, LDHA, MCT1, PKM2 and PFK1 could become potential biomarkers, either diagnostic or prognostic, in breast cancer, depending on the molecular subtypes and treatment involved.

### 2.2. Lung Cancer and Glucose Metabolism

LDHA and LDHB subunits play pivotal roles in the metabolic reprogramming of lung cancer cells. IHC analyses revealed elevated LDHA protein levels in both lung adenocarcinoma (LUAD) and lung squamous carcinoma (LUSC) compared to adjacent normal tissues [106]. This overexpression correlates with poor prognosis, radiotherapy resistance, enhanced glycolysis and hypoxia within the tumour microenvironment, as evidenced by positive associations between LDHA expression and markers such as HIF1A and GLUT-1. The overexpression of LDHA in non-small cell lung cancer (NSCLC) is primarily regulated by hypoxia-inducible factors (HIFs), which activate LDHA transcription under hypoxic conditions prevalent in tumours [106]. This regulatory mechanism underscores the enzyme’s contribution to the metabolic adaptations of cancer cells. Meanwhile, elevated LDHB levels in lung adenocarcinoma tissues are significantly associated with poor prognosis and reduced survival rates in cancer patients with KRAS-wild-type lung cancer [107]. Silencing LDHB in lung cancer cells has been shown to reduce invasion and migration capabilities, likely due to decreased intracellular glutathione levels and altered mitochondrial metabolism [107]. This highlights LDHB’s role in maintaining lung cancer cells’ redox balance and metastatic potential. The differential expression patterns of LDHA and LDHB in lung cancer suggest their potential as prognostic biomarkers.

Elevated expression of ALDOA has been observed in lung cancer patient samples of different subtypes, with studies indicating its association with tumour progression and poor prognosis. In an IHC analysis of NSCLC tumour samples, higher ALDOA levels correlate with increased metastatic potential and reduced survival rates [108]. Mechanistically, ALDOA promotes lung cancer metastasis by activating the HIF-1α/MMP9 axis, enhancing cell invasion and migration [108]. In lung adenocarcinoma, overexpression of ALDOA is linked to advanced tumour stages and unfavourable outcomes, suggesting its role as an independent prognostic factor [53]. In lung squamous cell carcinoma, ALDOA is significantly upregulated in metastatic tissues compared to non-metastatic and adjacent normal tissues, implicating its involvement in tumourigenesis and metastasis [50]. These findings highlight ALDOA’s potential as a biomarker for lung cancer progression and as a target for therapeutic intervention. Meanwhile, ENO1 has been implicated in the progression and metastasis of lung cancer. IHC and Western blot analyses have shown that ENO1 protein is overexpressed in lung cancer tissues, and associated with increased tumour growth, migration, and invasion [109,110,111]. Mechanistically, ENO1 interacts with hepatocyte growth factor receptor (HGFR) and activates HGFR and Wnt signaling pathways, leading to EMT and metastasis [110]. In NSCLC, ENO1 promotes cell proliferation through the focal adhesion kinase (FAK)-mediated PI3K/AKT pathway, further contributing to tumour progression [111]. These findings suggest that ENO1 plays a significant role in lung cancer development and may serve as a potential therapeutic target.

PFKP, PGAM1 and PGK1 are key metabolic enzymes in lung cancer, contributing to tumour progression and patient prognosis. PFKP was significantly overexpressed in lung cancer tissues as confirmed via IHC staining, which correlated with larger tumour size and poorer patient prognosis [112]. Functional studies confirmed that reducing PFKP expression in lung cancer cells decreased glucose uptake, lactate production, and cell proliferation, highlighting its role in tumour metabolism and growth. Furthermore, PGK1 was significantly upregulated in lung cancer tissues and associated with increased migratory and invasive capabilities of cancer cells [69]. Elevated PGK1 expression in IHC analysis was significantly correlated with poor outcomes in lung cancer patients [69]. Mechanistically, PGK1 promotes metastasis by interacting with HIV Tat-specific factor 1 (HTATSF1) involved in RNA metabolism, affecting multiple genes in tumour progression [69].

Several IHC studies on NSCLC tissues also reported that PGAM1 was significantly overexpressed compared to adjacent normal lung tissues, and associated with poor prognosis, increased tumour growth, and enhanced metastatic potential [113,114]. Li et al. demonstrated that PGAM1 functions as an oncogene by activating the transforming growth factor-β (TGF-β) signalling pathway in NSCLC [114]. PGAM1 enhances cancer cell proliferation and invasion by upregulating TGF-β signalling, contributing to tumour progression and EMT [114]. Another study revealed that PGAM1 is a critical regulator of mTOR-mediated tumour growth and is associated with aggressive NSCLC phenotypes [113]. PGAM1 promotes glycolytic metabolism, facilitating ATP production and biosynthetic processes essential for rapid tumour cell proliferation [113]. IHC analyses of NSCLC patient samples confirmed a significant correlation between high PGAM1 expression and poor survival [113]. The overexpression of PGAM1 in NSCLC patient samples and its roles in TGF-β and mTOR signalling pathways underscore its potential as a prognostic biomarker and therapeutic target.

In NSCLC, the protein expression levels of GLUT1, MCT1, and MCT4 have been extensively studied. An IHC study analysing 445 NSCLC patients demonstrated that those with positive expression of both GLUT1 and PKM2 (G+/P+) had significantly poorer overall and disease-free survival rates following curative R0 resection [115]. Elevated GLUT1 expression correlated with advanced tumour stage, lymphatic invasion, and pleural invasion [115]. These findings suggest that assessing GLUT1 and PKM2 expression could provide valuable prognostic information for NSCLC patients post-R0 resection. In addition, MCT1 protein was expressed in cancer cells and stromal cells within NSCLC tissues, as validated with tissue microarray and IHC staining [116]. Interestingly, MCT1 overexpression in cancer cells was associated with improved disease-specific survival, suggesting a less aggressive tumour phenotype [116]. Conversely, elevated MCT1 expression in stromal cells correlates with poorer prognosis, potentially due to its role in facilitating lactate uptake and promoting a supportive microenvironment for tumour growth [116]. Furthermore, MCT4 was upregulated in NSCLC tissue as shown by IHC staining, particularly in tumours exhibiting high glycolytic activity [117]. Elevated MCT4 protein levels have been linked to increased tumour aggressiveness and worse overall survival [117]. High MCT4 expression could be a negative prognostic marker in NSCLC patients [117].

### 2.3. CRC and Glucose Metabolism

The overexpression of glycolytic enzymes, glucose transporters, and monocarboxylate transporters in colorectal cancer (CRC) has been extensively documented. HK2 is frequently overexpressed in CRC tissues compared to normal colonic epithelium based on IHC studies [63,118,119]. This upregulation is associated with enhanced glycolytic activity, promoting tumour growth and survival under hypoxic conditions [118]. Elevated HK2 expression correlates with larger tumour size, deeper invasion, liver metastasis, and advanced TNM stages in CRC patients [63,118,119]. Moreover, high HK2 level is often associated with increased recurrence rates and overall mortality, establishing HK2 as an independent prognostic factor for both disease-free and overall survival in CRC [63,118,119]. Conversely, He et al. conducted IHC analysis of two independent cohorts using CRC tissue [120]. HK1 was overexpressed in 30% of CRC cases, and significantly correlated with advanced tumour stages and poorer overall survival [120]. Multivariate analysis further identified HK1 overexpression as an independent prognostic factor for CRC, suggesting that HK1 could serve as a valuable biomarker for predicting patient prognosis [120].

ENO1 was significantly upregulated in CRC tissues as reported in IHC staining, with higher expression levels correlating with advanced TNM stages, lymph node involvement, vascular invasion, and distant metastasis [58,121,122]. Elevated ENO1 expression was associated with poor overall survival in CRC patients, serving as an independent prognostic factor [58,121,122]. ENO1 enhances CRC cell proliferation, drug resistance, EMT, migration, and invasion by regulating oncogenic pathways, such as AMPK/mTOR signaling [58,121,122]. These findings suggest that ENO1 may serve as a valuable biomarker for CRC prognosis. Additionally, LDHB was downregulated in CRC tissue and associated with good prognosis, progression-free survival in CRC patients [44,81]. LDHB was suppressed by ribosomal protein RPS7 and Krüppel-like transcription factor 14 (KLF14) to inhibit glycolysis in CRC tissue [44,81].

PKM2 was significantly upregulated in CRC tissues as IHC staining and Western blot analyses reveal its predominant localization in the cytoplasm and nucleus, where it influences metabolic reprogramming and oncogenic signaling pathways, such as STAT3, β-catenin, and HIF-1α [123,124,125,126,127,128,129]. High PKM2 expression correlates with tumour grade, invasion depth, lymph node metastasis, and poor prognosis [123,124,125,126,127,128,129]. The dimeric form (TuM2-PK) of PKM2 is detectable in the serum or plasma of CRC patients and serves as a non-invasive biomarker [130,131,132]. Elevated serum/plasma PKM2 levels have been reported in CRC patients compared to healthy controls, showing potential for early detection and disease monitoring [130,131,132]. However, its diagnostic accuracy varies, requiring combination with other clinical biomarkers, such as CEA and CA19–9 to enhance sensitivity and specificity [130,131,132]. On the other hand, an IHC study observed that PGK1 expression was significantly higher in metastatic colon cancer tissues compared to non-metastatic ones [71]. PGK1 undergoes arginine methylation at residue R206 by protein arginine methyltransferase 1 (PRMT1), leading to increased phosphorylation at serine 203 [133]. These modifications enhance glycolytic activity and promote tumour growth in CRC cells. Elevated levels of PRMT1 and methylated PGK1 correlate with advanced tumour stages and poorer survival rates in CRC patients [133].

In CRC, several glycolytic transporters, namely, GLUT1, MCT1, and MCT4, exhibit altered expression in patients’ tissue and serum. Elevated GLUT1 in CRC tissues significantly correlated with adverse features, including poor differentiation, lymph node metastasis, and advanced tumour stages [134]. High GLUT1 expression in CRC tissue was associated with poorer overall and disease-free survival rates, underscoring its potential as a prognostic marker. Martins et al. presented a comprehensive IHC study highlighting the significance of MCT expression in various types of tissue samples among CRC patients, including primary tissue, lymph node and hepatic metastasis [135]. Their findings demonstrated that MCT1 and MCT4 were overexpressed in primary CRC tissues compared to adjacent normal tissues [135]. Specifically, MCT1 was overexpressed in primary tumours but a significant decrease in expression in lymph node and hepatic metastases [135]. Conversely, MCT4 was consistently overexpressed in primary tumours, lymph node metastases, and hepatic metastases, suggesting its role in facilitating metastatic progression [135]. The differential expression of these transporters correlated with various clinicopathological features. MCT1 expression was associated with older patient age, while MCT4 positivity correlated with tumour location in the colon and deeper tumour invasion [135]. Positive MCT1 expression in stage IV CRC tissue was linked to improved cumulative survival, whereas MCT4 expression was associated with poor prognosis [135]. These findings suggest that MCT1 and MCT4 are potential biomarkers for CRC progression and prognosis.

### 2.4. Liver Cancer and Glucose Metabolism

In hepatocellular carcinoma (HCC), several glycolytic enzymes and glucose transporters have been identified as potential biomarkers. Overexpression of GLUT1 and GLUT3 has been validated with IHC staining in HCC tissue samples, correlating with increased glucose uptake and glycolysis, thereby supporting rapid tumour growth [32,136,137]. Additionally, elevated GLUT3 expression was associated with poor prognosis in HCC patients [136]. A study involving IHC analysis of 157 HCC tumour samples reported that elevated HK2 levels were significantly associated with higher tumour grade and advanced cancer stage [138]. Increased HK2 expression correlated with reduced overall survival, suggesting its potential as a prognostic marker in HCC [138]. Another interesting investigation by Guzman et al. analysed HK2 expression in various types of liver tissues using IHC, including normal liver, non-dysplastic cirrhosis (NDC), liver cell dysplasia (LCD), and HCC [139]. Their study reported a progressive increase in HK2 protein expression from normal liver to NDC, LCD, and HCC. Elevated HK2 expression was also associated with more aggressive histological features in HCC, suggesting that HK2 upregulation may be an early event in hepatocarcinogenesis [66]. Thus, HK2 could serve as a diagnostic and prognostic biomarker in HCC.

LDH-C4 was significantly upregulated in HCC tissues compared to adjacent non-cancerous tissues in IHC staining, correlating positively with advanced clinical stages and larger tumour sizes [140]. HCC patients exhibiting high LDH-C4 expression demonstrated poorer prognosis, with reduced overall survival compared to those with low expression levels [140]. Meanwhile, an elevated preoperative serum LDH level was associated with poor overall survival and disease-free survival in HCC patients undergoing partial hepatectomy [141]. Multivariate analyses indicated that serum LDH could be an independent prognostic factor for both overall survival and disease-free survival in HCC [141]. On the other hand, among HCC patients treated with sorafenib, a high pre-treatment serum LDH level was associated with reduced progression-free survival and overall survival [142]. An increase in LDH levels after three months of treatment predicted poorer survival outcomes, suggesting that serum LDH could serve as a potential biomarker for treatment efficacy and disease progression [142]. These findings underscore the potential of LDH, particularly tissue-specific expression of LDH-C4 and elevated serum LDH level, for diagnosis, prognosis, and monitoring therapeutic responses in HCC.

Several IHC studies demonstrated that PKM2 was overexpressed in HCC tissues and associated with unfavourable clinicopathological features and poor patient prognosis [143,144,145,146]. HCC patients with high PKM2 expression exhibited significantly shorter survival times and a higher risk of recurrence compared to those with low PKM2 expression, highlighting its potential as a prognostic biomarker [143,144,145,146]. Meanwhile, MCTs also exhibit distinct expression patterns in HCC. Jeon et al. reported that MCT1 expression in HCC tissues varied depending on the tumour’s glucose uptake characteristics [147]. Specifically, MCT1 expression was higher in tumours with low [^18^F] FDG uptake but decreased in tumours with high [^18^F] fluorodeoxyglucose (FDG) uptake [147]. This suggests a potential inverse relationship between MCT1 expression and glycolytic activity in HCC tumours. Additionally, researchers investigated the expression of MCTs and GLUT1 in HCC samples [148]. MCT4 and GLUT1 expression increased progressively from non-neoplastic liver tissues to primary HCC and was highest in metastatic lesions [148]. This pattern suggests that elevated GLUT1 and MCT4 expression may play a role in tumour progression and metastasis in HCC. In contrast, MCT2 expression decreased progressively from non-neoplastic liver tissues to primary HCC and further in metastatic lesions [148]. This trend indicated that reduced MCT2 expression may be associated with HCC progression. The progressive decrease in MCT2 and increase in MCT4 expression from non-neoplastic tissues to metastatic lesions suggest that these transporters may serve as biomarkers for HCC progression and potential therapeutic targets.

### 2.5. Gastric Cancer and Glucose Metabolism

In gastric cancer (GC), several key enzymes and transporters, namely ALDOA, GLUTs, LDHA, and PKM2, have been widely investigated as potential biomarkers. ALDOA is frequently upregulated in GC tissues compared to adjacent normal tissues in IHC studies, associated with enhanced glycolytic activity, contributing to tumour progression [48,149,150]. Clinically, elevated ALDOA expression correlates with deeper tumour invasion, lymph node metastasis, and advanced clinical stages, while proteomic analysis suggests that ALDOA may be associated with gastric cancer stem cells (CSCs) [48,149,150,151]. ALDOA activates the epidermal growth factor receptor (EGFR) pathway, leading to downstream signaling that promotes GC cell proliferation and drug resistance [149]. Thus, ALDOA could serve as a valuable biomarker for prognosis and therapeutic monitoring in GC.

Several IHC studies demonstrated the overexpression of GLUT1 in GC tissues compared to normal gastric mucosa, which significantly correlated with tumour aggressiveness, invasion depth, lymph node metastasis, and advanced TNM stage [152,153,154]. Elevated GLUT1 expression was also significantly associated with short overall survival and disease progression in GC, suggesting the potential of GLUT1 as a poor prognostic biomarker [152,153,154]. In addition, another IHC study investigated the expression of GLUTs (1, 3, 6, and 10) in GC tissues and their association with clinical outcomes [155]. The findings revealed that all four GLUTs are expressed in GC tissue, with GLUT3 showing a significant correlation to both the UICC (Union for International Cancer Control) stage and overall survival in patients [155]. Overexpression of GLUT3 was associated with advanced tumour stages and poorer survival rates, suggesting its potential as a prognostic biomarker [155]. This study underscores the role of GLUTs in the metabolic reprogramming of gastric cancer cells, highlighting GLUT3 as a key player in tumour progression.

Yang et al. also reported the overexpression of GLUT3 in primary GC tumour and metastasis tumour tissues using IHC staining [156]. In depth analysis demonstrated that GLUT3 could promote histone modifications by regulating LDHA in GC, suggesting the possible interactions between glycolytic enzymes to facilitate non-metabolic activities in tumour progression [156]. Similarly, previous IHC studies have demonstrated the elevated LDHA expression in GC tissue compared to normal gastrointestinal mucosa, often correlated with advanced TNM staging, lymph node metastasis, poor prognosis, and low survival rates in GC patients [157,158,159,160]. Overexpression of LDHA in GC cells enhances glycolytic flux, contributing to tumour progression [157,158,159]. Elevated LDHA expression has been linked to poorer overall survival and disease-free survival in GC patients, suggesting its potential as a prognostic marker and therapeutic target. Additionally, PKM2 expression was significantly elevated in GC tissues and was mostly associated with poor prognosis and shorter survival, as shown in multiple IHC studies [74,161,162]. Notably, well-differentiated GC adenocarcinomas exhibited higher PKM2 expression compared to undifferentiated signet-ring cell GC carcinomas [162]. Elevated PKM2 expression in signet-ring cell gastric cancers correlated with shorter overall survival, suggesting its potential as an independent prognostic marker in this subtype [162]. Table 1 summarizes the key enzymes and proteins in the Warburg effect and glucose metabolism of the top five cancers.

## 3. Amino Acid Metabolism in Cancers

Amino acid metabolism is another key component of cancer metabolic reprogramming, supporting tumour growth, immune evasion, redox balance and drug resistance [163]. Cancer cells rewire amino acid pathways to meet increased bioenergetic and biosynthetic demands [163]. For instance, glutamine is the most abundant amino acid in plasma, in which different types of tumours rely on glutamine metabolism to facilitate the TCA cycle, nucleotide synthesis, and redox homeostasis [164]. Serine and glycine metabolism contribute to one-carbon metabolism which is essential for DNA synthesis and epigenetic modifications [165]. Branched-chain amino acids (BCAAs) activate mTORC1 signaling, promoting tumour proliferation [166]. Proline and arginine metabolism regulate oxidative stress and immune suppression, enhancing tumour progression [167,168]. Identifying biomarkers associated with dysregulated amino acid metabolism, such as amino acid transporters and critical enzymes, could improve cancer diagnosis, prognosis, and treatment strategies. Among these, ASCT2, GLS1, LAT1, SLC7A11, SLC3A2, and SHMT2 are key regulators of amino acid metabolism and have been implicated in tumour progression, therapy resistance, and immune evasion.

ASCT2 (SLC1A5) is a sodium-dependent neutral amino acid transporter that facilitates glutamine uptake, fuelling the TCA cycle and mTORC1 activation, making it essential for glutamine-addicted tumours [169]. Once inside the cell, glutaminase (GLS) converts glutamine to glutamate, which feeds into the TCA cycle and supports nucleotide and lipid biosynthesis [170]. GLS also maintains redox homeostasis by producing glutamate for glutathione synthesis, protecting cancer cells from oxidative stress [171]. Glutamate dehydrogenase (GDH) catalyses the reversible conversion of glutamate to α-ketoglutarate (α-KG), which is further utilised in the TCA cycle, contributing to energy production and biosynthesis in cancer cells [172]. SLC7A5 (LAT1) transports essential amino acids to activate mTORC1 signaling, promoting protein synthesis and proliferation [173]. SLC7A11 (xCT) enables cystine uptake for glutathione synthesis, protecting cancer cells from oxidative stress and ferroptosis [174]. SLC3A2, also known as CD98 heavy chain, is integral to amino acid transport and integrin signaling [175]. SLC3A2 acts as a chaperone for LAT1 and xCT, enhancing amino acid transport while supporting integrin-mediated tumour invasion [176]. SHMT2 catalyses serine-to-glycine conversion, sustaining nucleotide biosynthesis and redox homeostasis, especially in hypoxic tumours [177]. Overexpression of these regulators is linked to aggressive tumour behaviour, therapy resistance, and poor prognosis. Targeting these metabolic vulnerabilities through inhibitors or dietary interventions holds promise for cancer therapy by disrupting essential nutrient uptake and biosynthetic pathways.

### 3.1. Breast Cancer and Amino Acid Metabolism

Several studies have investigated the proteomic profiling of breast cancer tissues, revealing the subtype-specific patterns of amino acid transporters and metabolic enzymes, highlighting their potential role as prognostic biomarkers. A comprehensive proteomic analysis employing reverse-phase protein array (RPPA) technology on 289 breast cancer samples demonstrated higher ASCT2 and SHMT2 protein expression in aggressive subtypes, including HER2-positive and TNBC, compared to luminal subtypes [178]. Elevated ASCT2 and SHMT2 levels in these subtypes were associated with shorter recurrence-free survival, highlighting their potential as independent prognostic factors [178]. Kim et al. conducted another tissue microarray profiling of 702 breast cancer patients using IHC staining for glutamine metabolism-related proteins, including GLS1, GDH, and ASCT2, in stromal and tumoral sites [179]. HER2^+^ breast cancer had the highest expression of stromal GLS1, tumoral GDH, stromal GDH, and tumoral ASCT2, compared to TNBC and luminal types [179]. These elevated expressions suggest increased glutamine metabolic activity in HER2^+^ tumours. In addition, ASCT2 expression was inversely correlated with oestrogen receptor (ER) and progesterone receptor (PR) positivity [179]. Tumours negative for ER and PR showed higher ASCT2 expression, indicating increased glutamine uptake in these subtypes [179]. Collectively, these findings highlight the metabolic heterogeneity among breast cancer subtypes, with specific transporters and enzymes upregulated in more aggressive forms like HER2^+^ and TNBC.

TNBC is regarded as an aggressive subtype of breast cancer with limited choices of target therapies, while tumour-infiltrating lymphocytes (TILs) are often correlated with better outcomes in TNBC [180]. However, metabolic adaptations, particularly glutamine metabolism, may counteract the benefits of TILs [181]. The prognostic significance of GLS expression has been evaluated in node-positive TNBC patients with high TILs, which demonstrated that GLS overexpression correlated with worse disease-free survival (DFS) and overall survival (OS) [182]. Despite the presence of abundant TILs, patients with elevated GLS expression exhibited significantly poorer outcomes, suggesting that metabolic reprogramming through glutaminolysis undermines the protective role of immune infiltration [182]. GLS expression could serve as a negative prognostic biomarker in node-positive TNBC, even in cases with high TILs. In addition, Zhou et al. performed tissue microarray and discovered that the prognostic value of GLS1 in breast cancer patients varied based on H3K27me3 (trimethylation of histone H3 at lysine 27) expression and menopausal status [183]. High GLS1 expression was associated with worse prognosis, particularly in tumours with low H3K27me3 levels, suggesting epigenetic regulation of glutamine metabolism may influence tumour progression [183]. Postmenopausal patients with high GLS1 expression exhibited poorer survival, whereas premenopausal patients showed no significant correlation, indicating metabolic dependencies shift with hormonal changes [183]. GLS1 is a potential prognostic biomarker in breast cancer, but its impact is modulated by H3K27me3 expression and menopausal status. These findings underscore the importance of integrating metabolic and epigenetic profiling in breast cancer prognosis.

Oestrogen receptor-positive (ER+) breast cancer constitutes most breast cancer cases, with endocrine therapy being the standard treatment [184]. However, resistance to endocrine therapy remains a significant clinical challenge. Alfarsi et al. investigated the co-expression of SLC7A5 and SLC3A2 as potential predictors of response to endocrine therapy in ER+ breast cancer patients via IHC staining [185]. High co-expression of SLC7A5 and SLC3A2 in breast cancer tissue was significantly associated with aggressive tumour phenotypes, such as increased proliferation, invasion, and metastasis [185]. More importantly, among patients receiving endocrine therapy alone, those with SLC7A5+/SLC3A2+ tumours experienced poorer prognosis, higher recurrence and mortality rates [185]. Knockdown of SLC7A5 and SLC3A2 resulted in reduced proliferation of ER+ breast cancer cells and enhanced the sensitivity of ER+ breast cancer cells to tamoxifen [185]. The same group of researchers also investigated the co-expression of ASCT2 and glycolytic enzyme TALDO1 in ER+ luminal breast cancer [184]. Similarly, high expression of SLC1A5 and TALDO1 correlated with aggressive tumour features, including higher grade and increased proliferation [184]. Patients with high SLC1A5 expression experienced poorer outcomes following endocrine therapy, while the co-expression of SLC1A5 and TALDO1 was significantly associated with a higher risk of recurrence and mortality in breast cancer patients receiving endocrine therapy [184]. Previous IHC studies by Ansari et al. also demonstrated that the high expression of SLC3A2 and SLC7A5 in TNBC, HER2^+^ and luminal B subtypes was significantly associated with poor patient outcome and shorter survival especially in patients with ER+ breast tumours [186,187]. Thus, the co-expression of ASCT2, SLC7A5 and SLC3A2 may serve as potential biomarkers for identifying ER+ highly proliferative subclass and HER2^+^ subclass of breast cancer patients at higher risk of poor response to endocrine therapy.

Serine hydroxymethyltransferases (SHMTs) are pivotal enzymes in one-carbon metabolism, facilitating the reversible conversion of serine to glycine and contributing to nucleotide biosynthesis [188]. Two primary isoforms exist: SHMT1, located in the cytoplasm, and SHMT2, found in mitochondria [188]. In breast cancer, previous IHC studies demonstrated that SHMT2 protein was significantly overexpressed in breast cancer tissues compared to noncancerous tissues, which correlated with increased tumour aggressiveness, including advanced TNM staging and higher Elston grade [189,190]. Higher SHMT2 expression was observed in grade III tumours compared to grades I–II, suggesting its role in tumour progression [189]. Elevated SHMT2 expression has been linked to poorer prognosis in breast cancer patients [178,189,190]. Notably, SHMT2’s prognostic value appears more significant in ER- breast cancer cases, indicating its potential as a biomarker for this subgroup [190]. SHMT2 promotes breast cancer cell proliferation through the activation of vascular endothelial growth factor (VEGF) and mitogen-activated protein kinase (MAPK) signaling pathways [191]. By regulating serine/glycine metabolism, SHMT2 supports nucleotide biosynthesis and redox balance, which are essential for rapidly proliferating cancer cells [191]. The association between SHMT2 overexpression and poor prognosis underscores its potential as a prognostic biomarker in breast cancer management.

### 3.2. Lung Cancer and Amino Acid Metabolism

Argininosuccinate synthase 1 (ASS1) is a key enzyme in the urea cycle, facilitating the synthesis of arginine from citrulline and aspartate to support cellular metabolism [168]. Its expression patterns in lung cancer tissues have been the subject of various studies, revealing potential implications for tumour behaviour and patient prognosis. ASS1 was significantly overexpressed in NSCLC tissue samples based on IHC staining [192]. The absence of ASS1 expression was associated with increased angiogenesis, suggesting that ASS1 deficiency may contribute to tumour progression [192]. Moreover, the presence of ASS1 in tumour cells correlated with a better prognosis in NSCLC patients, especially when cancer-associated fibroblasts (CAFs) did not express arginase-2 (ARG2) [192]. This association underscores the potential of ASS1 as a prognostic biomarker in lung cancer. Additionally, several amino acid transporters, namely ASCT2, LAT1 and SLC3A2, are dysregulated in lung cancer, contributing to tumour growth, survival, and metabolic reprogramming.

In lung cancer, ASCT2 exhibits distinct expression patterns across various histological subtypes, primarily overexpression in NSCLC subtypes. Hassanein et al. demonstrated that SLC1A5 was overexpressed in both adenocarcinoma (ADC) and squamous cell carcinoma (SCC) subtypes of NSCLC when compared to normal lung tissues in IHC analysis [193]. Further analysis revealed that ASCT2 was predominantly localized to the cytoplasmic membrane of cancer cells [193]. ASCT2 expression was significantly higher in SCC compared to ADC, while male patients also expressed higher SLC1A5 compared to female patients [193]. Most of the glutamine transport in lung cancer cells is sodium-dependent, with ASCT2 accounting for about half of this activity, highlighting the prognostic value of ASCT2 in supporting cancer cell metabolism and proliferation [193,194]. Another study by Shimizu et al. also supported that ASCT2 was significantly overexpressed in NSCLC, which correlated with advanced disease stage, lymphatic permeation, vascular invasion, increased cell proliferation, angiogenesis, and mTOR phosphorylation [195]. These associations were particularly pronounced in adenocarcinoma patients [195]. Furthermore, validation with an independent cohort confirmed that ASCT2 serves as an independent marker for poor outcomes in lung adenocarcinoma patients [195].

Yazawa et al. investigated the co-expression of ASCT2 and LAT1 in lung adenocarcinoma [196]. Their findings indicated that high expression levels of both transporters were significantly associated with tumour aggressiveness and poor survival outcomes [196]. This co-expression suggests a coordinated role between these two proteins in facilitating tumour growth and progression. In addition, IHC analysis of 295 NSCLC cases revealed that LAT1 protein was detected in the cytoplasm and/or on the plasma membrane of tumour cells [197]. Plasma membrane expression of LAT1 was associated with tumour histology, differentiation grade, pathologic stage, T classification, pleural invasion, lymph-vessel invasion, and overall survival rate [197]. Imai et al. also investigated different subtypes of stage I NSCLC tissues, which demonstrated that LAT1 expression was more prominent in squamous cell carcinoma and large cell carcinoma, compared to adenocarcinoma [198]. A significant correlation was observed between LAT1 expression and the Ki67 labelling index, indicating a link to proliferative activity [198]. The 5-year survival rate for LAT1-positive patients was significantly lower compared to LAT1-negative patients [198]. Multivariate analysis confirmed that positive LAT1 expression was an independent factor for predicting poor prognosis in these patients [198]. These findings suggest that LAT1 expression could serve as a valuable prognostic marker in NSCLC cases.

Elevated serum levels of SLC3A2 have been detected in patients with lung adenocarcinoma (LUAD) and lung squamous cell carcinoma (LUSC), which also corresponded to the increased mRNA level of SLC3A2 in cancer tissues [199]. This overexpression was associated with enhanced tumourigenesis via the MEK/ERK signaling pathway, underscoring the importance of SLC3A2 in cancer cell proliferation and survival [199]. High SLC3A2 expression also correlated with poorer overall survival in LUAD patients, suggesting its potential as a non-invasive prognostic biomarker [199]. SLC3A2 influences the tumour microenvironment by promoting the polarization of tumour-associated macrophages (TAMs) toward the M2 phenotype, which supports tumour growth [200]. This effect is mediated through metabolic reprogramming involving arachidonic acid pathways [200]. Taken together, SLC3A2 is significantly overexpressed in lung cancer tissues and patient serum, highlighting its potential as a diagnostic and prognostic biomarker.

Serine metabolism plays a critical role in cellular function by contributing to biosynthetic processes, redox balance, and signaling pathways [201]. Serine is a non-essential amino acid involved in nucleotide biosynthesis, lipid metabolism, and methylation reactions [165]. Many cancer cells rely on de novo synthesis through the serine synthesis pathway (SSP) to support rapid proliferation and survival [165]. Phosphoglycerate dehydrogenase (PHGDH) is the rate-limiting enzyme involved in the SSP, catalysing the conversion of 3-phosphoglycerate to 3-phosphohydroxypyruvate [201]. A study involving 319 NSCLC patients demonstrated significantly increased PHGDH expression at both mRNA and protein levels in tumour tissues compared to adjacent non-tumour tissues [202]. IHC analysis revealed that high PHGDH expression was significantly associated with lymph node metastasis and advanced TNM stage [202]. Moreover, elevated PHGDH levels correlated with poorer 5-year overall survival rates, suggesting its potential as a prognostic marker in NSCLC [202]. Additionally, another study involving bioinformatics analysis of 720 LUAD patients and tissue microarray analysis of 75 LUAD samples indicated that high PHGDH expression defines a metabolic subtype characterized by enhanced glycolysis and increased serine production from glucose [203]. This metabolic reprogramming supports rapid cell proliferation and migration by facilitating nucleotide biosynthesis [203]. PHGDH overexpression has been linked to resistance against the tyrosine kinase inhibitor erlotinib in LUAD cell lines, with inhibition of PHGDH re-sensitizing resistant cells to treatment [203]. While PHGDH is significantly overexpressed in NSCLC and LUAD subtypes, its regulatory mechanisms and functional roles underscore its potential as a prognostic marker in lung cancer management.

Serine metabolism may play an important role in lung cancer progression. Previous studies demonstrated that SHMT1 was significantly overexpressed in lung cancer tissues, which was associated with increased cell proliferation and tumour progression [204]. Knockdown experiments of SHMT1 in lung cancer cell lines led to cell cycle arrest and p53-dependent apoptosis [204]. This suggests that SHMT1 plays a crucial role in maintaining the proliferative capacity of lung cancer cells. Moreover, SHMT2 was significantly upregulated in lung adenocarcinoma tissues, and associated with advanced stages of lung cancer, suggesting its involvement in tumour aggressiveness and potential as a prognostic marker [205]. While both SHMT1 and SHMT2 are overexpressed in lung cancer tissues, their utility as independent prognostic markers remains uncertain, which warrants further validation in a larger cohort.

### 3.3. CRC and Amino Acid Metabolism

Numerous IHC and tissue microarray studies have reported elevated GLS expression in CRC tissues compared to adjacent normal tissues, indicating its role in the metabolic adaptation of CRC cells to support rapid growth and survival [171,206,207,208,209]. The overexpression of GLS is particularly evident in highly proliferative regions of the tumour, such as invasive margins and areas with tumour budding [207]. This suggests that GLS may support the invasive properties of CRC by providing energy and metabolites needed for cell motility and invasion. High GLS expression correlated with advanced tumour stages, lymph node metastasis, and poor prognosis in CRC patients [171,206,207,208,209]. The upregulation of GLS in CRC tissues reflects its important role in maintaining the metabolic flexibility of cancer cells, supporting their growth and survival. This makes GLS a promising biomarker in CRC. On the other hand, GDH was significantly overexpressed in CRC tissues and metastatic lesions, which correlated with advanced tumour stages and poor differentiation in IHC staining [210]. High GDH expression was also significantly associated with poorer overall survival and disease-free survival rates in CRC patients, while multivariate analyses identified GDH expression as an independent prognostic factor [210]. Functionally, GDH promoted CRC cell proliferation, migration, and invasion via STAT3-mediated EMT, while knockdown of GDH inhibited these processes [210]. The findings suggest that GDH could be a prognostic biomarker for identifying cancer metastasis in CRC patients.

Protein expression of a few amino acid transporters has been investigated in CRC clinical samples. SLC3A2 was significantly overexpressed in CRC tissues based on IHC staining and associated with enhanced tumour growth and metastasis [211]. Functionally, macrophage migration inhibitory factor (MIF) bound directly to SLC3A2, enhancing its expression and subsequently promoting CRC progression [211]. The MIF/SLC3A2 axis activated the AKT/GSK-3β signaling pathway, leading to increased tumour cell proliferation and metastasis [211]. The overexpression of SLC3A2 in CRC tissues and its involvement in critical signaling pathways highlight its potential as a biomarker and therapeutic target in CRC. Meanwhile, the upregulation of SLC1A5 in CRC tissues was significantly associated with enhanced tumour growth and survival [212,213]. SLC1A5-mediated glutamine uptake supports the metabolic demands of rapidly proliferating CRC cells, contributing to tumour growth and survival [213]. More importantly, Toda et al. found a significant correlation between KRAS mutational status and SLC1A5 expression based on tissue microarray, when elevated SLC1A5 levels were notably associated with tumour depth and vascular invasion in KRAS-mutant CRC cases [212]. Knockdown of SLC1A5 suppressed the migration of KRAS-mutant CRC cells, indicating that SLC1A5 could be a novel therapeutic target against KRAS-mutant CRC [212]. Interestingly, clinical samples from CRC patients resistant to cetuximab exhibited significantly higher SLC1A5 expression in IHC staining compared to those responsive to the treatment, suggesting the potential of SLC1A5 as a prognostic biomarker to predict chemo-response in CRC [214]. These findings highlight the critical role of SLC1A5 as a potential biomarker in CRC cases, particularly with KRAS mutations or resistance to cetuximab.

SLC7A5 was significantly overexpressed in early-stage (stages I and II) CRC cases, although it was less prevalent in tumours with mucinous histology and those exhibiting lymphovascular invasion in IHC staining [215]. Elevated SLC7A5 expression correlated with improved OS and DFS in early-stage CRC patients, especially stage II patients, where high expression was an independent indicator of better clinical outcomes [215]. SLC7A5 was also expressed in activated lymphocytes, including NK, T, and B cells [215]. Its overexpression in early-stage CRC may enhance anti-tumour immune responses, contributing to improved patient outcomes [215]. SLC7A5 overexpression could serve as a favourable prognostic marker in early-stage CRC. Similarly, another study by Najumudeen et al. reported that KRAS-mutant CRCs exhibited increased SLC7A5 expression, suggesting a link between oncogenic KRAS signaling and amino acid transporter regulation [216]. SLC7A5 facilitated glutamine consumption in CRC cells to sustain the metabolic demands of rapid proliferation [216]. By maintaining intracellular amino acid levels, SLC7A5 promotes bulk protein synthesis, underpinning the enhanced proliferation of KRAS-mutant CRC cells [216]. SLC7A5 plays a critical role in the metabolic reprogramming and progression of CRC, particularly in the context of KRAS mutations.

In contrast, SLC25A21 expression was significantly reduced in KRAS-mutant CRC tissues based on IHC staining, while patients with lower SLC25A21 expression exhibited poorer survival outcomes, particularly in KRAS-mutant CRC cases [217]. SLC25A21 is a mitochondrial carrier involved in transporting 2-oxoadipate and α-ketoglutarate across the inner mitochondrial membrane [218]. Reduced SLC25A21 expression led to decreased efflux of α-ketoglutarate from mitochondria, enhancing glutamine replenishment to support the metabolic demands of rapidly proliferating KRAS-mutant CRC cells [217]. The increased glutamine metabolism resulting from SLC25A21 downregulation provides substrates for GTP synthesis, maintaining persistent KRAS activation and promoting tumour progression [217]. Downregulation of SLC25A21 was correlated with increased invasion, migration, metastasis and drug resistance of KRAS-mutant CRC cells [217]. Restoring SLC25A21 expression reduced KRAS signaling and sensitized CRC tumours to cetuximab, suggesting that targeting SLC25A21 could enhance therapeutic efficacy [217]. Thus, SLC25A21 could serve as an independent prognostic marker for metabolic intervention strategies in CRC.

Since KRAS mutations are found in 40 to 50% of CRC cases, identifying the expression levels of SLC1A5, SLC7A5 and SLC25A21 could help stratify patient risk [219,220]. SLC1A5 (ASCT2) is mostly upregulated, facilitating glutamine uptake to support KRAS-driven tumour growth, making it a prognostic and therapeutic biomarker [212]. SLC7A5 (LAT1) is also overexpressed in CRC tissue, promoting mTOR activation and tumour aggressiveness, with high expression correlating with poor survival and resistance to EGFR inhibitors [216]. In contrast, SLC25A21 is downregulated in CRC tissue, enhancing glutamine metabolism and sustaining KRAS activity, with low expression linked to poor prognosis and cetuximab resistance [217]. Collectively, these transporters offer valuable insights into CRC pathophysiology, and their combined evaluation could improve risk stratification, therapy selection, and personalized treatment strategies in KRAS-mutant CRC. Further clinical validation is required to establish their diagnostic, prognostic, and predictive utility in routine practice.

### 3.4. Liver Cancer and Amino Acid Metabolism

Kidney-type GLS1 and liver-type GLS2 play distinct roles in HCC. GLS1 is frequently upregulated in HCC tissues, while in contrast, GLS2 expression is often reduced in HCC. A study involving 1140 HCC patients demonstrated that GLS1 expression had a sensitivity of 74.6% and specificity of 84.2% for diagnosing HCC [221]. When combined with glypican 3 (GPC3) and alpha-fetoprotein (AFP), the diagnostic performance improved, achieving a sensitivity of 84.6% and specificity of 88.6% [221]. Additionally, high GLS1 expression correlated with advanced tumour stages and poorer disease-free survival, underscoring its prognostic value [221]. Another IHC investigation reported that GLS1 was highly expressed in HCC tissues but minimally present in non-cancerous liver tissues [222]. This differential expression suggests that GLS1 could serve as a reliable marker for distinguishing HCC from non-cancerous liver conditions [222]. In contrast to GLS1, GLS2 expression was typically diminished in HCC, which could be attributed to the CpG methylation of GLS2 promoter [223]. Further research is warranted to explore the utility of these proteins for early detection and therapeutic strategies in HCC.

GDH1 expression was significantly upregulated in HCC tissues compared to adjacent non-cancerous liver tissues in IHC studies [224]. This upregulation supports the notion that GDH1 contributes to tumourigenesis by promoting glutaminolysis, which sustains cellular survival under conditions of glucose deprivation [224]. In addition, glutamine synthetase (GS) is an ATP-dependent enzyme that catalyses the conversion of glutamate and ammonia to glutamine [225]. Elevated GS expression was observed in HCC tissues and patient serum, which was associated with tumour invasion and metastasis [226]. In vitro and in vivo studies demonstrated that GS facilitated HCC cell migration and invasion through EMT modulation [226]. Notably, GS demonstrates high diagnostic accuracy for alpha-fetoprotein (AFP)-negative HCC cases, with an area under the receiver operating characteristic curve of 0.913 [226]. Combining GS with AFP enhances diagnostic sensitivity and specificity, suggesting its value in early detection of HCC. Further research is warranted to fully validate the potential of GDH and GS as early biomarkers in HCC.

Asparagine synthetase (ASNS) is an enzyme that catalyses the ATP-dependent conversion of aspartate and glutamine into asparagine and glutamate [208]. This reaction is crucial for protein synthesis, nucleotide biosynthesis, and cellular response to amino acid deprivation [208]. IHC studies have demonstrated that ASNS expression is significantly elevated in HCC tissues and associated with various clinicopathological features, including serum AFP levels, tumour size, microscopic vascular invasion, tumour encapsulation, TNM stage, and Barcelona Clinic Liver Cancer (BCLC) stage [227]. Notably, patients exhibiting low ASNS expression levels have been observed to have poorer overall survival, suggesting that ASNS serves as an independent prognostic factor for OS in HCC patients [227]. On the other hand, glutamic-oxaloacetic transaminase 2 (GOT2) is a mitochondrial enzyme that plays a pivotal role in amino acid metabolism and the malate-aspartate shuttle [228]. This shuttle is essential for transferring reducing equivalents across the mitochondrial membrane, thereby linking cytosolic and mitochondrial metabolic pathways [228]. GOT2 expression is often downregulated in HCC tumour tissues in IHC studies and associated with advanced disease progression and poorer patient prognosis [229]. In vivo studies further demonstrate that the tumour-suppressive role of GOT2 in HCC is linked to its influence on glutamine metabolism [229]. Loss of GOT2 leads to enhanced glutaminolysis, nucleotide synthesis, and glutathione production [229]. This reprogramming supports the cellular antioxidant system, thereby activating the PI3K/AKT/mTOR signaling pathway, which contributes to HCC progression [229]. Therefore, assessing GOT2 expression levels could provide valuable prognostic information in HCC.

The expression levels of ASCT2 and LAT1 were significantly upregulated in HCC tissues and correlated with larger tumour size, poorer histological differentiation, and advanced tumour staging in IHC analysis [32,230]. Elevated levels of ASCT2 and LAT1 are also associated with reduced overall survival, suggesting their potential as independent prognostic factors in HCC [32,230]. Meanwhile, xCT (SLC7A11) is frequently overexpressed in HCC tissues, contributing to tumour development and metastasis [231,232,233]. The elevated expression of xCT is also significantly associated with the overexpression of HIF1α, programmed death ligand 1 (PD-L1), colony-stimulating factor 1 (CSF1), tumour-associated macrophage (TAM) and myeloid-derived suppressor cell (MDSC) infiltration [231,232]. Functionally, xCT promotes PD-L1 and CSF1 expression through αKG-HIF1α cascade, resulting in intratumoral TA and MDSC infiltration and regulation of ferroptosis and the tumour microenvironment [231,232]. High expression of xCT also correlates with advanced tumour stage and unfavourable survival in HCC patients, especially those with strong PD-L1, suggesting that xCT may serve as an independent prognostic marker in HCC [231,233].

### 3.5. Gastric Cancer and Amino Acid Metabolism

In GC, GLS2 expression was significantly downregulated in cancer tissues compared to adjacent non-cancerous tissues as confirmed in IHC staining [234]. This reduction in GLS2 correlated with enhanced tumour cell proliferation and migration, alongside decreased apoptosis [234]. Overexpression of GLS2 suppressed these malignant behaviours, implying a tumour-suppressive function for GLS2 in GC [234]. Further research is warranted to elucidate whether GLS2 could serve as a potential biomarker for diagnostic or prognostic purposes in GC [234]. Moreover, GDH was significantly overexpressed in GC tissue compared to the normal gastric mucosa tissues based on IHC analysis, while it was primarily expressed in the cytoplasmic membrane of GC cells [235]. The expression of GDH in GC tissue was closely related to the rate of CD34 positive, cancer cell differentiation degree, infringement of capsular, vascular tumour emboli, lymph node metastasis, and nerve infiltration [235]. More importantly, elevated expression of GDH was correlated with better prognosis and longer survival in GC patients, suggesting its potential as an independent prognostic biomarker [235].

ASCT2 was significantly overexpressed in GC tissues compared to adjacent non-cancerous gastric mucosa, as shown by IHC and Western blotting [236,237]. ASCT2 was predominantly localized to the cell membrane of gastric carcinoma cells, facilitating efficient glutamine uptake essential for tumour metabolism [236]. Silencing ASCT2 expression significantly reduced the proliferation of gastric cancer cell lines, underscoring its role in tumour growth [237]. The overexpression of ASCT2 in GC tissues and its involvement in key metabolic pathways highlight its potential as a biomarker. In addition, ASS1 expression is significantly elevated in GC tissues compared to normal gastric mucosa in IHC studies [238,239]. ASS1 influences autophagic processes in GC cells [239]. High ASS1 protein expression in GC tissue is positively correlated with aggressive tumour phenotype and unfavourable prognosis, supporting the utility of ASS1 as a useful prognostic marker for GC [238,239].

Meanwhile, IHC studies have demonstrated that LAT1 is significantly overexpressed in GC cells, which is predominantly localized to the plasma membranes of carcinoma cells [240,241]. High LAT1 expression in GC tissues has been significantly associated with adverse clinicopathologic features, including larger tumour size, lymph node metastasis, deeper local invasion, and advanced TNM stage [241]. Downregulation of LAT1 expression in GC cell lines has been shown to inhibit cell proliferation and induce cell cycle arrest in the G0/G1 phase [240]. Patients with high LAT1-expressing non-scirrhous GC have been reported to have a significantly poorer prognosis compared to those with low LAT1 expression [241]. Multivariate analysis has identified LAT1 expression as an independent prognostic factor in this subgroup [241]. Furthermore, IHC studies have shown that xCT is significantly overexpressed in GC tissues compared to adjacent normal tissues [242]. This overexpression is associated with enhanced tumour growth and poor prognosis [242]. Functionally, xCT activates the PI3K/AKT signaling pathway to inhibit ferroptosis and support GC progression by enhancing cell proliferation, migration, and invasion [242]. Table 2 summarizes the key enzymes and proteins in the amino acid metabolism of the top five cancers.

## 4. Lipid Metabolism

Lipid metabolism plays a pivotal role in cancer metabolic reprogramming by fuelling energy production, promoting membrane biosynthesis, and enhancing survival under stress [20]. Cancer cells often alter several key enzymes and transporters involved in lipid uptake, synthesis, and oxidation to meet their energetic and biosynthetic needs. Unlike normal cells, which primarily obtain fatty acids (FAs) from dietary sources, cancer cells enhance de novo fatty acid synthesis by modulating ACLY, ACC, FASN and SCD [20]. ATP citrate lyase (ACLY) converts citrate to acetyl-CoA as precursor for lipid synthesis, while acetyl-CoA carboxylase (ACC) catalyses the carboxylation of acetyl-CoA to malonyl-CoA [243]. Fatty acid synthase (FASN) is a rate-limiting enzyme in FA synthesis, while stearoyl-CoA desaturase-1 (SCD1) converts saturated fatty acids to monounsaturated fatty acids (MUFAs), promoting membrane fluidity and tumour survival [33,244]. Cancer cells upregulate lipid transporters, namely CD36 (FAT) and fatty acid-binding proteins (FABPs) to increase fatty acid uptake [245,246]. Fatty acid oxidation (FAO) provides ATP, NADH, and FADH_2_ to support oxidative phosphorylation in mitochondria, which key enzymes involved are namely carnitine palmitoyltransferase 1 (CPT1A) and acyl-CoA dehydrogenases (ACADs) [243]. CPT1A facilitates FA transport into mitochondria, while ACADs catalyse FA oxidation in the mitochondria [243].

### 4.1. Breast Cancer and Lipid Metabolism

Several studies have investigated the expression of lipid metabolism-related proteins across different breast cancer subtypes and metastatic sites, providing insights into their potential roles in tumour behaviour and patient prognosis [247,248]. Kim et al. analysed the expression of lipid metabolism-related proteins in 476 invasive breast cancer cases of these four different subtypes using tissue microarray and IHC staining [247]. The proteins examined included perilipin 1 (PLIN1), CPT1A, FASN, FABP4, and acyl-CoA oxidase 1 (ACOX1). Notably, HER2-enriched tumours exhibited the highest expression of these proteins, highlighting that HER2^+^ breast tumours demonstrate higher metabolic plasticity with elevated lipid metabolism compared to other subtypes [247]. ACOX-1 positivity was associated with significantly shorter overall survival in all the subtypes, while particularly FABP4 positivity was significantly associated with shorter disease-free survival and overall survival in TNBC. These observations suggest that ACOX-1 and FABP4 are poor prognostic biomarkers in metastatic breast cancer, which can be specific to certain subtypes.

Another IHC study by Cha et al. compared the expression profiles of lipid metabolism-related proteins between invasive lobular carcinoma (ILC) and invasive ductal carcinoma (IDC) [249]. ILC and IDC are the two most common histological subtypes of breast cancer. IDC is the most common type of breast cancer (70–80% of cases) originating from the epithelial cells of the milk ducts, whereas ILC arises from the epithelial cells of the lobules (milk-producing glands), accounting for 10–15% of breast cancers [78,250]. ILC is more often ER+ and PR+ but HER2^-^, whereas IDC has more heterogeneous receptor expression, with some cases being HER2^+^ or TNBC [78,250]. Their findings indicated that hormone-sensitive lipase (HSL) and FABP4 were more frequently expressed in ILC compared to IDC, particularly in Luminal A-type ILC [249]. Conversely, PLIN1 was more commonly expressed in IDC than in ILC [249]. These differences suggest distinct lipid metabolic pathways between these histological subtypes, which may influence tumour behaviour and response to therapy. Hormone therapy is more effective in ILC than in IDC, but chemotherapy is less effective in ILC [250]. IDC can be more aggressive, especially HER2^+^ and triple-negative subtypes, requiring more aggressive treatment [250].

In addition, Jung et al. investigated 149 cases of metastatic breast cancer to determine the expression of lipid metabolism-related proteins across various metastatic sites, including bone, brain, liver, and lung, using IHC staining [248]. Their analysis revealed that FASN expression varied significantly according to molecular subtype, with higher expression in HER2^+^ types and lower expression in Luminal A and TNBC subtypes [248]. Additionally, PLIN1 positivity was identified as an independent poor prognostic factor, associated with shorter overall survival [248]. These results highlight the potential of lipid metabolism-related proteins as biomarkers and therapeutic targets in metastatic breast cancer. Furthermore, the same team of researchers also investigated the expression patterns of lipid metabolism-related proteins in phyllodes tumours of the breast and their associations with tumour grade and patient prognosis [251]. Phyllodes tumours (PTs) are rare fibroepithelial breast tumours that originate from the periductal stromal cells rather than from the epithelial components of the breast [252]. These tumours account for less than 1% of all breast tumours and can exhibit a wide spectrum of biological behaviour, ranging from benign to highly aggressive malignant forms [252]. Higher-grade PTs exhibited increased stromal expression of HSL, perilipin 2, FABP4, CPT-1, and FASN [251]. This suggests that lipid metabolism alterations are associated with tumour aggressiveness. Perilipin 2, FABP4, CPT-1, and FASN were associated with shorter overall survival in PT patients [252]. Notably, stromal perilipin 2 positivity emerged as an independent prognostic factor for reduced disease-free survival. These findings highlight the potential role of lipid metabolism-related proteins as prognostic biomarkers in PT progression. Collectively, these studies underscore the heterogeneity of lipid metabolism-related protein expression across breast cancer subtypes and their potential implications for prognosis and targeted therapy. High expression of FASN also significantly correlated with HER2^+^ status and brain metastasis, suggesting FASN could serve as a potential biomarker for distinguishing HER2-enriched breast cancer and predicting metastasis at later stage of malignancy. High expression of PLIN also positively correlated with poor prognosis, particularly in HER2^+^ tumours and TNBC, which warrants further research to validate its clinical significance in these breast cancer subtypes.

### 4.2. Lung Cancer and Lipid Metabolism

Lipid metabolism alterations are increasingly recognized as significant contributors to lung cancer pathogenesis, with distinct expression patterns observed across different histological subtypes. Lung adenocarcinoma (LUAD) is a predominant subtype of NSCLC that exhibits distinct alterations in lipid metabolism, with specific proteins playing pivotal roles in tumour development and progression [253]. A proteomic analysis of primary LUAD tumours using global mass spectrometry identified the upregulation of enzymes involved in fatty acid synthesis and oxidation, underscoring their potential role as early biomarkers [254]. Key lipid metabolism-related proteins, such as FASN and ACC, showed elevated expression in LUAD tissues compared to normal lung tissue [254]. These enzymes are crucial for de novo lipogenesis in cancer cells to meet increased lipid demands for membrane synthesis and energy production. Additionally, IHC studies have demonstrated increased nuclear expression of SREBP1 in lung adenocarcinoma (LUAD) tissues compared to normal lung tissues [253]. This upregulation correlates with enhanced lipogenesis and tumour growth, indicating its role in lung cancer metabolism [253]. Proteomic analyses using LC-MS have identified higher expression levels of ACSL1, ACSL4, and ACSL5 significantly correlated with LUAD [255]. ACSL5 expression was inversely associated with Ki67 in low-grade tumours, while ACSL3 was positively associated with Ki67 in high-grade tumours [255]. These findings from patient-derived samples emphasize the critical role of lipid metabolism-related proteins in LUAD pathogenesis. The consistent upregulation of enzymes like FASN and ACC suggests their potential as biomarkers for diagnosis and as targets for therapeutic intervention in LUAD. A recent LC-MS/MS proteomic analysis on small cell lung cancer (SCLC) tissue samples identified two distinct categories of primary tumours based on their protein expression profiles, revealing the importance of lipid metabolism, particularly phospholipid metabolism, and immune response pathways in SCLC metastasis [256]. Both high-density and low-density lipoprotein cholesterol levels were found to be independent prognostic factors for disease-free survival in SCLC patients [256]. High stromal expression of phospholipase A2 group IIA (sPLA2-IIA) was associated with delayed disease recurrence in patients with limited-stage SCLC [256]. The expression patterns of lipid metabolism-related proteins vary notably between NSCLC and SCLC, offering insights into tumour biology and potential clinical applications. Given the growing evidence that phospholipid remodelling plays a critical role in tumour biology and cancer metabolism, integrating phospholipidomic profiling with enzyme-focused analyses offers a promising avenue for future research. Such multi-omics approaches could provide a more comprehensive understanding of the metabolic reprogramming in lung cancer, potentially uncovering novel biomarkers and therapeutic targets.

### 4.3. CRC and Lipid Metabolism

IHC analysis of CRC specimens revealed higher ACLY staining scores in cancerous tissues, with even greater expression observed in metastatic cases [257]. A positive correlation between ACLY and β-catenin (CTNNB1) protein levels has been identified, suggesting a collaborative role in CRC progression [257]. ACLY stabilizes β-catenin to promote its nuclear translocation and enhance its transcriptional activity, thereby facilitating CRC cell migration and invasion [257]. High ACLY expression correlates with increased risk of lymph node and distant metastasis, poorer tumour differentiation, and advanced clinical stages [258]. Patients with elevated ACLY levels exhibit higher recurrence rates and reduced overall survival [258]. These findings underscore the potential of ACLY as a biomarker for CRC aggressiveness and as a target for therapeutic intervention.

Acetyl-CoA synthetase short-chain family member 2 (ACSS2) is an enzyme that catalyses the conversion of acetate into acetyl-CoA, playing a crucial role in lipid biosynthesis and energy metabolism [243]. IHC analyses have demonstrated that ACSS2 protein expression is significantly higher in CRC tissues compared to normal colorectal tissues [259]. ACSS2 exhibits distinct subcellular localization patterns in benign versus malignant tissues. In benign tissues, ACSS2 immunoreactivity is predominantly diffuse within the cytosol [259]. In contrast, CRC samples often display localized cytosolic staining, with a lower cytosolic/nuclear ratio, suggesting a preferential nuclear presence of ACSS2 in cancer cells [259]. Current research on tissue-based analyses highlights the need for further investigation into developing ACSS2 as a potential biomarker for CRC diagnosis and prognosis.

On the other hand, FASN protein levels are markedly increased in CRC tissues compared to adjacent normal tissues in IHC staining [260,261,262,263,264]. This overexpression correlates with advanced tumour stages, lymph node metastasis, and poorer patient prognosis [262,264]. Mechanistic studies suggest that FASN may modulate the AMPK/mTOR and Wnt signaling pathways, contributing to tumour progression, cell proliferation, invasion, and metastasis [260,261]. Additionally, CRC patients have significantly higher FASN concentrations in the serum compared to healthy individuals [265]. Elevated serum FASN levels are associated with tumour extent, lymph node involvement, distant metastasis, and advanced clinical stages [265]. High serum FASN levels have been identified as an independent predictor of reduced overall and disease-free survival rates in CRC patients [265]. In summary, FASN protein is overexpressed in CRC tissues and elevated in patient serum, correlating with disease progression and poorer outcomes. These findings underscore the potential of FASN as both a diagnostic and prognostic biomarker in CRC.

In CRC, differential expression of specific ACSL isoforms has been observed, influencing tumour behaviour and patient outcomes. Overexpression of ACSL1 and ACSL4 has been linked to enhanced tumour aggressiveness [266]. In a tissue microarray study of 77 stage II CRC patients, simultaneous overexpression of ACSL1, ACSL4, and stearoyl-CoA desaturase (SCD) correlated with poorer disease-free survival, suggesting a cooperative role in promoting EMT and metastasis [266]. In contrast, low ACSL5 expression in CRC tissues is associated with early tumour recurrence [267]. In an IHC study involving 72 patients, those with reduced ACSL5 levels experienced significantly increased tumour recurrence within one year, independent of TNM and UICC stages [267]. Collectively, dysregulated expression of ACSL1, ACSL4 and ACSL5 isoforms in CRC tissues is associated with tumour progression and patient outcomes, warranting additional research for biomarker development.

The expression of various FABPs has been studied in CRC tissues and patient serum, revealing significant associations with disease progression and potential diagnostic implications. Zhang et al. have demonstrated that FABP4 is highly expressed in CRC tissues and patient serum, which correlates with advanced tumour stages and lymph node metastasis [268]. Notably, FABP4 levels in tumour tissues are inversely related to E-cadherin expression and positively associated with Snail protein levels as shown in IHC studies, suggesting a role in promoting EMT and tumour progression [268]. Similarly, FABP6 expression is significantly upregulated in CRC tissues compared to adjacent normal tissues [269]. The increased presence of FABP6 in tumour cells indicates its potential involvement in CRC carcinogenesis [269]. Increased serum levels of FABP6 have also been observed in CRC patients [269]. Notably, serum concentrations of both FABP4 and FABP6 significantly decreased in two weeks post-surgery, suggesting their potential utility in monitoring treatment response [269]. When combined with CEA, the sensitivity and specificity of FABP4 and FABP6 have been improved, suggesting that a panel of these biomarkers could enhance CRC detection [269]. The overexpression of FABP4 and FABP6 in CRC tissues and their elevated levels in patient serum are significantly associated with disease progression and metastasis [268,269]. These findings underscore the potential of FABP4 and FABP6 as biomarkers for CRC diagnosis, prognosis, and monitoring therapeutic responses. Further research is warranted to validate these biomarkers in larger CRC patient cohorts.

### 4.4. Liver Cancer and Lipid Metabolism

In HCC, key enzymes and transporters involved in de novo lipogenesis, fatty acid uptake, and cholesterol synthesis, such as FASN, SCD1, SREBPs, and fatty acid transport proteins, are frequently dysregulated in tumour tissues, contributing to tumour growth, immune evasion and therapy resistance. FASN protein expression is notably elevated in HCC tissue compared to adjacent non-cancerous liver tissues [270]. This upregulation is associated with enhanced tumour cell proliferation and progression [270]. ACLY is also significantly upregulated in HCC tissues and associated with poor prognosis and advanced tumour stages [271]. Functionally, ACLY contributes to HCC progression by activating the Wnt/β-catenin signaling pathway, thereby promoting cancer stemness and metastasis [271].

On the other hand, FABP4 and FABP5 have been implicated in HCC tumour progression and patient prognosis as reported by several IHC and tissue microarray studies. FABP4 expression is significantly reduced in HCC tissues compared to adjacent normal tissues [272]. Lower FABP4 levels are associated with larger tumour size, presence of portal vein tumour thrombus (PVTT), and shorter OS and DFS [272]. In contrast, overexpression of FABP5 in HCC tissues is associated with poorer tumour differentiation, vascular invasion, advanced TNM stages, and reduced OS and DFS, suggesting its importance as a prognostic marker in HCC [273,274]. Interestingly, elevated serum FABP5 levels in HCC patients are significantly associated with postoperative survival, independent of its tissue expression levels [273]. Although FABP4 and FABP5 demonstrate opposite expression levels in HCC tissues and serum, these proteins are promising biomarkers and therapeutic targets in HCC, as their dysregulation reflects the metabolic dependency of liver cancer on fatty acid synthesis and utilization.

ACSL3 and ACSL4 play significant roles in HCC development and progression. Their expression patterns in tumour tissues have prognostic implications and may serve as potential therapeutic targets. Ndiaye et al. assessed the expression and localization of ACSL3 and ACSL4 in HCC, cholangiocarcinoma (CCA), and hepatic metastases by performing IHC analyses on tissue microarrays and subcellular fractionation of HepG2 cells [275]. ACSL3 and ACSL4 exhibited increased expression in HCC tissues compared to normal liver. ACSL4 expression was significantly higher in HCC than in all other tumour types, while ACSL3 expression was similar in HCC and hepatic metastases. Increased ACSL3 expression distinguished HCC from CCA, while combined ACSL3 and ACSL4 staining scores differentiated HCC from hepatic metastases. ACSL4 localized to the plasma membrane, lipid droplets, and endoplasmic reticulum (ER), while ACSL3 was associated with lipid droplets and the ER. Taken together, the differential expression and localization of ACSL3 and ACSL4 can aid in distinguishing HCC from other hepatic tumours, suggesting their potential as diagnostic biomarkers.

Several studies also highlight the clinical significance of ACSL4 in HCC. High ACSL4 expression is associated with poor survival in HCC patients, particularly those treated with postoperative adjuvant transarterial chemoembolization (TACE) [276,277,278,279]. High ACSL4 expression promotes HCC progression by stabilizing the oncoprotein c-Myc through the ERK/FBW7/c-Myc axis [276,279]. Additionally, patients with high ACSL4 expression in pre-treatment tumour tissues also exhibited complete or partial responses to sorafenib, which is a first-line treatment for advanced HCC [280]. These findings underscore the potential of ACSL4 as a biomarker to predict sorafenib responsiveness in HCC patients [280]. Clinically, ACSL4 was positively correlated with the expression of SREBP1 in HCC patients [276]. Another IHC study by Li et al. revealed that SREBP1 was significantly upregulated in HCC tissues compared to adjacent non-cancerous liver tissues, which was associated with larger tumour size, higher histological grade, and advanced TNM stage [281]. Patients with elevated SREBP1 levels have poorer overall and disease-free survival rates, suggesting the prognostic potential of this protein in HCC [281]. Functionally, SREBP1 enhances tumour cell proliferation, migration, and invasion, suggesting its role in promoting HCC progression [281].

SCD1 is significantly overexpressed in HCC tissues, which correlates with enhanced tumour growth, metastasis, immune infiltration and poor clinical outcomes [282]. Elevated serum levels of SCD1 have been observed in HCC patients, suggesting its potential as a non-invasive biomarker for HCC diagnosis and prognosis [282]. Chen et al. have further demonstrated the diagnostic accuracy of serum SCD1 levels in distinguishing HCC patients from healthy individuals, validating its utility in clinical settings [282]. SCD1 overexpression in HCC tissues and elevated serum levels underscore its significance as a biomarker for disease progression and a potential therapeutic target. Meanwhile, SLC27A5 (FATP5) was significantly downregulated in the IHC analysis of HCC tissues [283,284]. This downregulation is linked to DNA hypermethylation of the SLC27A5 promoter region [283,284]. Lower SLC27A5 levels correlate with poorer patient prognosis, including larger tumour size and higher recurrence rates [283,284]. Functionally, SLC27A5 deficiency leads to increased lipid peroxidation, activating the NRF2/TXNRD1 pathway, which contributes to tumour progression and resistance to therapies like sorafenib [283,284]. In summary, the dysregulated expression of ACLY, ACSL3, ACSL4, FABP4, FABP5, SCD1, SLC27A5, and SREBP1 in HCC underscores their critical roles in lipid metabolism alterations contributing to tumour development and progression. These proteins hold potential as biomarkers for prognosis and as targets for therapeutic intervention in HCC.

### 4.5. Gastric Cancer and Lipid Metabolism

In GC, FASN overexpression has been consistently reported in cancer tissues compared to adjacent normal mucosa [285,286,287,288]. This upregulation correlates with advanced TNM stage, lymph node metastasis, poor differentiation, and increased tumour invasiveness [285,286,287]. Clinically, high FASN protein expression is an unfavourable prognostic indicator, as it is associated with reduced OS and DFS in GC patients [285,286,287,288]. Similarly, elevated serum levels of FASN have been observed in GC patients compared to healthy controls [289]. Notably, increased serum FASN levels were detected even in early-stage gastric cancer patients, suggesting its utility as a non-invasive biomarker for early detection [289]. ACLY is also significantly overexpressed in GC tissues compared to adjacent non-cancerous tissues in IHC studies [290,291,292]. High ACLY expression correlates with advanced tumour stages and lymph node metastasis, indicating its role in tumour aggressiveness [290,291,292]. Elevated ACLY expression has been associated with poorer overall survival in GC patients, suggesting its potential as a prognostic biomarker [290,291,292]. On the other hand, CD36 protein expression in GC tissue has been linked to tumour progression and metastasis. Studies using IHC staining have shown that higher CD36 expression correlates with poorer prognosis in GC patients [293]. Additionally, hypoxia-induced CD36 expression has been observed in peritoneal metastases, suggesting its role in fatty acid uptake and tumour spread [294]. CD36 expression is associated with increased migration and invasion of GC cells, particularly in adipocyte-rich environments [294]. This highlights its potential as a biomarker for aggressive disease and a therapeutic target for intervention.

Studies have demonstrated that SCD1 is significantly overexpressed in GC tissues compared to adjacent non-cancerous tissues [295]. This upregulation is associated with enhanced tumour growth, migration, and resistance to ferroptosis [295]. High SCD1 expression levels in GC tissues also correlate with poor patient prognosis, suggesting its potential as a prognostic biomarker [295]. In addition, CPT1A is significantly upregulated in GC tissues, which correlates with enhanced tumour proliferation, invasion, and EMT [296]. Elevated CPT1A expression is significantly associated with advanced TNM stages, lymph node metastasis, and poorer overall survival in GC patients [296]. IHC analyses have demonstrated higher CPT1A levels in tumours with distant metastasis and *Helicobacter pylori* positivity [296]. Moreover, studies have reported increased CPT1A levels in the serum of GC patients compared to healthy controls [297]. This elevation is associated with poor prognosis and lymph node metastasis, suggesting its potential as a non-invasive biomarker for GC diagnosis and prognosis [297]. The overexpression of CPT1A in GC tissues and its elevated levels in patient serum underscore its pivotal role in tumour metabolism and progression. Its association with adverse clinical outcomes positions CPT1A as a promising biomarker and potential therapeutic target in gastric cancer management.

The overexpression of ACC1 has been reported in GC tissue using IHC staining, underscoring its importance in tumour progression [298]. ACC1 upregulation in GC tissue is associated with enhanced fatty acid synthesis, promoting tumour growth and progression [298]. More importantly, increased expression of phosphorylated ACC1 (p-ACC1) has been identified as an independent prognostic factor in gastric cancer patients without lymph node metastasis, indicating its potential role in monitoring tumour aggressiveness [298]. Meanwhile, lipoprotein lipase (LPL) is a key enzyme in lipid metabolism, hydrolysing triglycerides in circulating lipoproteins to release free fatty acids for cellular uptake [243]. LPL is significantly overexpressed in GC tissues, especially in cases with lymph node metastasis [299]. This overexpression facilitates increased lipid uptake, supporting tumour growth and metastatic potential [299]. Mechanistically, leptin, an adipokine often elevated in obesity, suppresses the cleavage of LPL, leading to enhanced lipid uptake and promoting lymph node metastasis in GC [299]. This highlights a link between metabolic disorders and cancer progression. More importantly, alterations in serum lipid profiles, such as decreased high-density lipoprotein cholesterol (HDL-C) and increased low-density lipoprotein cholesterol (LDL-C), have been observed in GC patients, which may indirectly reflect changes in LPL activity [300]. Further research is needed to elucidate the potential of LPL as a non-invasive biomarker in GC through serum level assessments.

Peroxisome proliferator-activated receptors (PPARs) are nuclear hormone receptors that regulate lipid metabolism, inflammation, and cellular differentiation. The three isoforms, including PPARα, PPARδ/PPARβ, and PPARγ, exhibit distinct expression patterns and functional roles in GC. PPARδ is overexpressed in approximately 90% of GC tissues, including both intestinal and diffuse subtypes, while its expression is low or absent in normal gastric epithelium [301]. Elevated PPARδ expression correlates with increased tumour cell proliferation, invasion, and metastasis [301]. It interacts with the Hippo pathway coactivator YAP1 to promote SOX9 expression, contributing to tumourigenesis [301]. Similarly, Ma et al. revealed significantly higher PPARγ expression in GC tissues compared to adjacent non-cancerous and normal gastric mucosa [302]. Activation of PPARγ has been shown to inhibit gastric cancer cell proliferation, migration, and invasion [302]. It downregulates β-catenin signaling and may induce apoptosis, suggesting a tumour-suppressive role [302]. The expression patterns and functional implications of PPARα in GC remain less defined. Further research is needed to elucidate the roles of PPARs in GC fully and to assess their potential as non-invasive biomarkers. Table 3 summarizes the key enzymes and proteins in the amino acid metabolism of top five cancers.

## 5. Metabolic Biomarkers in Clinical Trials and Applications

Validation in prospective clinical trials is essential for biomarkers to reach clinical implementation. Companion diagnostic development must meet regulatory requirements set by agencies like the United States Food and Drug Administration (FDA) and the European Medicines Agency (EMA), focusing on assay standardization, analytical reproducibility, and clinical utility [303]. Despite the vast array of metabolic proteins and enzymes identified as being dysregulated in cancers, only a limited number have advanced to clinical application. For metabolic biomarkers to reach this stage, these molecules must demonstrate not only mechanistic relevance and diagnostic accuracy but also reproducibility and clinical utility in prospective studies [304]. Several metabolic biomarkers, mainly those involved in glycolysis, have been approved or are currently undergoing evaluation in clinical trials for cancer detection, prognosis, or treatment monitoring.

### 5.1. Tumour M2-Pyruvate Kinase (TuM2-PK)

One of the most extensively studied metabolic biomarkers in clinical settings is TuM2-PK, an isoform of pyruvate kinase M2 (PKM2) secreted by tumour cells. TuM2-PK is elevated in the plasma or stool of patients with various cancers, including breast, CRC, gastric, lung, and pancreatic cancers [130,305,306,307,308]. Commercial assays, such as the ScheBo^®^ Tumour M2-PK Test, (Schebo Biotech AC, Giessen, Germany), have been approved in Europe and used clinically to aid CRC detection via stool or plasma samples [309]. Studies report increased sensitivity when TuM2-PK is combined with carcinoembryonic antigen (CEA) or faecal occult blood testing (FOBT), making it valuable in early detection and post-surgical monitoring [130,309,310].

### 5.2. Lactate Dehydrogenase (LDH)

Serum LDH level is considered a nonspecific marker of tissue damage and is elevated in many conditions, including cancers, haemolysis, myocardial infarction, and liver disease. In oncology, elevated serum LDH may be used as a prognostic indicator or for monitoring disease progression or response to therapy, especially in lymphoma and melanoma [311,312,313]. Although nonspecific, its elevation often correlates with high tumour burden and aggressive disease phenotypes. In solid tumours such as CRC, HCC and NSCLC, serum LDH has prognostic utility, especially in the context of hypoxic or highly glycolytic tumours [141,314,315]. Moreover, pre-treatment LDH levels have been used to stratify cancer patients for targeted therapies like immunotherapy and tyrosine kinase inhibitors [316,317].

### 5.3. Isocitrate Dehydrogenase 1 (IDH1) and 2-Hydroxyglutarate (2-HG)

IDH1 mutations are among the most studied metabolic alterations in cancers, particularly in gliomas, acute myeloid leukaemia, cholangiocarcinoma, and chondrosarcoma [318,319,320,321]. These mutations confer a neo-morphic enzymatic activity that converts alpha-ketoglutarate to the oncometabolite 2-HG, which accumulates at high levels in tumour cells [322]. Elevated 2-HG induces widespread epigenetic changes, contributing to tumourigenesis through aberrant DNA and histone methylation [320]. Clinically, 2-HG serves as a valuable biomarker for IDH1-mutant tumours and can be detected in tissues and, to a lesser extent, in blood or cerebrospinal fluid using advanced techniques such as magnetic resonance spectroscopy and mass spectrometry [319,320,322]. Importantly, inhibitors targeting mutant IDH1 that reduce 2-HG levels show promise in preclinical and clinical settings, highlighting the therapeutic and diagnostic significance of this metabolic axis in cancer [323,324].

### 5.4. Liquid Biopsy Approaches and Analytical Challenges in Clinical Settings

Liquid biopsy has emerged as a transformative tool in cancer diagnostics, offering minimally invasive access to circulating tumour components such as cell-free DNA (cfDNA), circulating tumour cells (CTCs), and extracellular vesicles. This approach enables real-time monitoring of tumour dynamics, early detection, and therapeutic response assessment, making it highly valuable for clinical chemists and cancer researchers. Despite promising advances, several analytical challenges remain critical for successful clinical translation. Sample handling is a paramount concern, as pre-analytical variables such as blood collection tubes, processing times, storage conditions, and cfDNA isolation methods significantly influence biomarker integrity and assay reproducibility [325,326,327]. Standardization of protocols is essential to minimize variability and ensure reliable downstream analyses. Assay standardization is another challenge due to the diverse technologies used, including PCR-based methods, digital droplet PCR, and next-generation sequencing. Each platform varies in sensitivity, specificity, and quantification accuracy. Establishing consensus on quality control measures, reference materials, and reporting standards is vital to facilitate inter-laboratory comparability and regulatory acceptance. Thus, overcoming these challenges through rigorous method validation and harmonization efforts will be crucial for integrating liquid biopsy into routine clinical practice, thereby enhancing personalized oncology care.

## 6. Future Directions

Emerging technologies in spatial metabolomics and single-cell metabolomics are revolutionizing our understanding of metabolic heterogeneity within tumours, which is essential in tumour progression, tumour microenvironment, and therapeutic response. Spatial metabolomics enables the precise localization of metabolites directly within tissue sections, preserving the native architectural context of the tumour and its microenvironment [328,329]. This approach uncovers metabolic gradients, niches, and spatial interactions among cancer cells, stromal cells, and immune infiltrates not seen in bulk tissue analyses [329]. Single-cell metabolomics complements this by providing detailed metabolic profiles at the resolution of individual cells [328]. This high-resolution approach reveals previously unrecognized cellular heterogeneity and dynamic metabolic states within tumours, clarifying the metabolic plasticity that underlies phenotypic diversity, metastasis, and drug resistance [330]. The integration of spatial and single-cell metabolomics holds great promise for advancing biomarker discovery, enabling the identification of cell-type specific metabolic vulnerabilities and spatially defined metabolic signatures that can inform patient stratification and guide precision oncology interventions [330,331,332,333]. As these technologies continue to evolve, they are poised to transform cancer research and clinical diagnostics by linking metabolism with cellular identity and spatial context.

## 7. Conclusions

The intricate relationship between cancer metabolism and disease progression has become increasingly apparent, with metabolic reprogramming now recognized as a hallmark of cancer. This review comprehensively highlights how key metabolic enzymes, transporters, and proteins involved in glucose, amino acid, and lipid metabolism are differentially expressed across the top five cancers worldwide, namely lung, breast, CRC, liver, and gastric cancers. These molecules not only support tumour proliferation, survival, and therapy resistance but also show considerable promise as diagnostic, prognostic, and predictive biomarkers. Glycolytic enzymes such as LDHA, PKM2, HK2, and ENO1, along with nutrient transporters like GLUT1, ASCT2, and LAT1, are consistently upregulated across multiple cancer types and have been linked to poor prognosis and therapy resistance. Similarly, enzymes involved in glutamine metabolism (e.g., GLS1, GDH) and lipid biosynthesis (e.g., FASN, ACLY) further underscore the metabolic dependencies that distinguish cancer cells from normal counterparts. These findings not only support the role of metabolic proteins as potential tissue or serum-based biomarkers but also highlight their value as therapeutic targets in the era of precision oncology. Despite significant progress, few metabolic biomarkers have reached clinical implementation. Only a handful, such as TuM2-PK and serum LDH, are currently utilized in diagnostic or therapeutic settings. This gap between discovery and clinical application underscores the urgent need for rigorous validation strategies, including large-scale multi-omics integration, prospective biomarker trials, and standardization of assay platforms. In conclusion, advancing our understanding of cancer-specific metabolic rewiring and translating this knowledge into clinically actionable biomarkers will be essential for enhancing early detection, personalizing treatment strategies, and improving outcomes across high-burden malignancies. Future efforts must focus on refining biomarker validation pipelines, leveraging artificial intelligence to mine complex metabolic datasets, and developing combination strategies that co-target metabolic vulnerabilities alongside conventional therapies.

## Figures and Tables

**Table 1:** Key enzymes and proteins in the Warburg effect and glucose metabolism.

Protein/Enzyme	Function in Glycolysis/Metabolism	Cancer Types	Biomarker Role
ALDOA	Cleaves fructose-1,6-bisphosphate into triose phosphates	Breast, Lung, CRC, Gastric	Prognostic, EMT, invasion
ENO1	Catalyzes conversion of 2-PG to PEP	Breast, Lung, CRC, Gastric	Prognostic, chemoresistance, stemness
GLUT1/GLUT3/GLUT4	Facilitates glucose uptake	Breast, CRC, Lung, Gastric	Diagnostic, prognostic, associated with aggressiveness
HK2/HK1	Initiates glycolysis via phosphorylation of glucose	Breast, CRC, Liver, Lung	Prognostic, linked to HIF-1α, recurrence
LDHA/LDHB	Converts pyruvate to lactate; supports NAD+ regeneration	Breast, Lung, CRC, Gastric, Liver	Prognostic, treatment monitoring, serum biomarker (LDHA)
MCT1/MCT4	Lactate and pyruvate export	Breast, CRC, Lung	Prognostic, therapy resistance, hypoxia response
PFKP	Rate-limiting conversion of F6P to F1,6BP	Breast, Lung	Prognostic, therapy resistance
PGAM1	Converts 3-PG to 2-PG	Breast, Lung	Prognostic, migration, EMT
PGK1	Generates ATP via 1,3-BPG to 3-PG conversion	CRC, Lung	Prognostic, promotes angiogenesis, metastasis
PKM2	Catalyzes PEP to pyruvate; allows accumulation of glycolytic intermediates	Breast, Lung, CRC, Gastric, Liver	Prognostic, metastasis, therapy response, serum marker
SGLT1	Sodium-dependent glucose transporter	HER2^+^ Breast	Prognostic, linked with HER2 stabilization
TPI1	Isomerizes DHAP and G3P	Breast, CRC	Prognostic, proliferation, invasion

**Table 2:** Key enzymes and proteins in the amino acid metabolism.

Protein/Enzyme	Function in Amino Acid Metabolism	Cancer Types	Biomarker Role
ASCT2 (SLC1A5)	Glutamine transporter, supports mTOR activation	Breast, CRC, Lung, Gastric, Liver	Prognostic, therapy resistance (e.g., anti-EGFR)
GDH (GLUD1)	Converts glutamate to α-KG	CRC, Gastric, Liver	Prognostic, metastasis, links to EMT
GLS1/GLS2	Converts glutamine to glutamate	Breast, CRC, Liver	Prognostic, therapeutic target (GLS inhibitors)
GOT2	Links amino acid metabolism to TCA cycle	Liver	Tumour suppressor, poor prognosis when lost
LAT1 (SLC7A5)/CD98 (SLC3A2)	Transports essential amino acids (e.g., leucine), mTOR activation	Breast, CRC, Gastric, Liver, Lung	Prognostic, survival marker, endocrine resistance
xCT (SLC7A11)	Cystine/glutamate antiporter, regulates redox and ferroptosis	Gastric, Liver	Prognostic, immune escape, therapeutic target
SHMT1/SHMT2	Converts serine to glycine (one-carbon metabolism)	Breast, Lung	Prognostic, linked to hypoxia and cell proliferation
PHGDH	Catalyzes serine biosynthesis from 3-PG	Lung, Breast	Prognostic, therapy resistance, metabolic reprogramming

**Table 3:** Key enzymes and proteins in lipid metabolism.

Protein/Enzyme	Function in Lipid Metabolism	Cancer Types	Biomarker Role
FASN	De novo fatty acid synthesis from acetyl-CoA	Breast, Lung, CRC, Prostate	Prognostic, therapeutic target (FASN inhibitors)
ACLY	Converts citrate to acetyl-CoA, fuels lipogenesis	Breast, Lung	Prognostic, linked to growth and metastasis
ACC	Converts acetyl-CoA to malonyl-CoA	Breast, Liver	Prognostic, therapeutic target, FA synthesis regulation
SCD1	Converts saturated FA to monounsaturated FA	Breast, Lung, CRC	Prognostic, linked to membrane fluidity and survival
CPT1A	Controls fatty acid oxidation (FAO)	Breast, Prostate	Prognostic, metabolic plasticity, therapy resistance
FABP5/FABP4	Fatty acid-binding proteins aiding lipid uptake	Prostate, Breast	Prognostic, associated with aggressiveness
LPL	Hydrolyzes triglycerides for fatty acid uptake	Prostate, Breast	Prognostic, lipid-driven tumour progression

## Data Availability

No new data were created or analyzed in this study.

## Abbreviations

The following abbreviations are used in this manuscript:

2-HG	2-hydroxyglutarate
ACC	Acetyl-CoA carboxylase
ACAD	Acyl-CoA dehydrogenase
ACOX	Acyl-coenzyme A oxidase 1
ACLY	ATP citrate lyase
ACSL	Acyl-CoA synthetase long-chain
ACSS	Acyl-CoA synthetase short-chain
ADP	Adenosine diphosphate
AFP	Alpha-fetoprotein
AKT	Protein kinase B
ALDOA	Aldolase A
AMPK	AMP-activated protein kinase
ARG2	Arginase-2
ASCT2	Alanine serine cysteine transporter 2 (SLC1A5)
ASNS	Asparagine synthetase
ASS1	Argininosuccinate synthetase 1
ATP	Adenosine triphosphate
BCAT1	Branched-chain amino acid transaminase 1
BRCA	Breast cancer gene
CAF	Cancer-associated fibroblast
CD36	Cluster of differentiation 36
CEA	Carcinoembryonic antigen
CPT1A	Carnitine palmitoyltransferase 1A
CRC	Colorectal cancer
CSF	Cerebrospinal fluid
CSF1	Colony-stimulating factor 1
CTNNB1	Catenin beta 1
DBC	Ductal breast carcinoma
EMA	European Medicines Agency
EMT	Epithelial–mesenchymal transition
ENO1	Alpha-enolase 1
EGCG	Epigallocatechin gallate
EGFR	Epidermal growth factor receptor
ER	Endoplasmic reticulum
FA	Fatty acid
FABP	Fatty acid-binding protein
FAO	Fatty acid oxidation
FASN	Fatty acid synthase
FAT	Fatty acid translocase
FATP	Fatty acid transporter
FBP1	Fructose-1,6-bisphosphatase 1
FDA	Food and Drug Administration
FDG	Fluorodeoxyglucose
FOBT	Faecal occult blood test
FOXM1	Forkhead box protein M1
G6PD	Glucose-6-phosphate dehydrogenase
GC	Gastric cancer
GDH (GLUD1)	Glutamate dehydrogenase
GLS1/2	Glutaminase 1/2
GLUT1	Glucose transporter 1
GOT2	Glutamic-oxaloacetic transaminase 2
GS	Glutamine synthetase
GSH	Glutathione
HCC	Hepatocellular carcinoma
HGFR	Hepatocyte growth factor receptor
HIF-1α	Hypoxia-inducible factor 1-alpha
HK2	Hexokinase 2
HTATSF1	HIV Tat-specific factor 1
IDC	Invasive ductal carcinoma
IHC	Immunohistochemistry
ILC	Invasive lobular carcinoma
IL-1β	Interleukin-1 beta
LAT1	L-type amino acid transporter 1 (SLC7A5)
LDH/LDHA/LDHB	Lactate dehydrogenase A/B
LPL	Lipoprotein lipase
MAPK	Mitogen-activated protein kinase
MCT1	Monocarboxylate transporter 1
MUFA	Monounsaturated fatty acid
mTOR	Mechanistic target of rapamycin
MYC	MYC proto-oncogene
NAD+/NADH	Nicotinamide adenine dinucleotide (oxidized/reduced)
NADPH	Nicotinamide adenine dinucleotide phosphate (reduced)
NGS	Next-generation sequencing
NSCLC	Non-small cell lung cancer
OXPHOS	Oxidative phosphorylation
PD-L1	Programmed death-ligand 1
PDH	Pyruvate dehydrogenase
PDK1	Pyruvate dehydrogenase kinase 1
PFK1	Phosphofructokinase-1
PFKP	Phosphofructokinase, platelet,
PGAM1	Phosphoglycerate mutase 1
PGK1	Phosphoglycerate kinase 1
PHGDH	Phosphoglycerate dehydrogenase
PI3K	Phosphoinositide 3-kinase
PKM2	Pyruvate kinase M2
PLIN1	Perilipin1
PPARs	Peroxisome proliferator-activated receptors
PPP	Pentose phosphate pathway
PTM	Post-translational modification
PTs	Phyllodes tumours
PVTT	Portal vein tumour thrombus
ROS	Reactive oxygen species
SCD1	Stearoyl-CoA desaturase 1
SGLT1	Solute carrier family 5 member 1
SHMT1/2	Serine hydroxymethyltransferase 1/2
SLC	Solute carrier
SREBPs	Sterol regulatory element-binding proteins
TALDO	Transaldolase
TAM	Tumour-associated macrophage
TCA	Tricarboxylic acid (cycle)
TME	Tumour microenvironment
TNM	Tumour, Node, Metastasis (staging)
TP53	Tumour protein p53
TPI1	Triosephosphate isomerase
TuM2-PK	Tumour M2-pyruvate kinase
VEGF	Vascular endothelial growth factor
UICC	Union for International Cancer Control
xCT	Cystine/glutamate antiporter (SLC7A11)
YAP	Yes-associated protein
